# Mapping the landscape of psychological literature on threat from 1961 to 2023 through structural topic modeling

**DOI:** 10.1371/journal.pone.0350996

**Published:** 2026-06-05

**Authors:** Huixiang Ouyang, Ching Wan, Ronald Fischer

**Affiliations:** 1 Nanyang Technological University, Singapore, Singapore; 2 D’Or Institute for Research and Education (IDOR), Rio de Janeiro, Brazil; University of Padova, ITALY

## Abstract

The past decades have generated a substantial volume of psychological literature on threat. However, the absence of systematic cross-field synthesis has resulted in limited understanding of major research domains and relationships between different lines of threat research. We analyzed 51,903 psychological publications on threat retrieved from APA PsycInfo, Scopus, and Web of Science Core Collection that were published between 1961 and 2023. We conducted structural topic modeling on publication titles and abstracts to identify key research topics, and network analysis on the resulting topics to map the thematic structure of the literature. 25 topics emerged, organized into four thematic areas through exploratory graph analysis: 1) threat processing mechanisms, 2) health and clinical threats, 3) social psychological threats, and 4) collective threats. Network analysis revealed differential connectivity patterns within and between thematic areas. Areas showed limited connectivity with each other and no area emerged as a central hub, suggesting gaps in cross-domain integration. Topic prevalence trends revealed diversification in research interest over time, together with responsiveness to broader developments within psychology and evolving societal concerns. Notably, mechanism-focused research declined over the past decade while event-driven research on specific threats increased, indicating reactive rather than theory-driven investigation. These findings provide insights into the landscape of psychological literature on threat and reveal critical gaps in current examinations alongside strategic opportunities to advance cross-field integration.

## Introduction

Humanity contends with threat across diverse situations and contexts, ranging from fleeting moments in everyday life when immediate dangers demand rapid responses like a near-miss car accident, to situations where threat persists for a prolonged period such as the recent COVID-19 pandemic. Given the ubiquity of threat in the human experience, what has psychology as a field offered to the understanding of threat and what should we offer? To answer these questions, we need to first evaluate the landscape of existing psychological research on threat, which spans across decades and subfields. For instance, clinical psychologists have investigated the role of threat perceptions in different psychological disorders [1,2], cognitive psychologists have examined attentional processes involved in threat perception [3,4], and social psychologists have studied socially focused threats such as intergroup threat [5] and stereotype threat [6].

To date, no known review has been conducted on the existing research on threat across psychology as a field. Past qualitative reviews and meta-analyses of psychological research on threat have only synthesized findings within subfields pertaining to specific topics, populations, or methodologies of interest. Two key gaps in our present understanding of the psychological literature on threat arise from the lack of a comprehensive, cross-field synthesis. Firstly, researchers lack clarity on the key research efforts on threat across different subfields, which is crucial to guide the identification of important but underexplored research questions concerning the psychology of threat. Secondly, researchers lack clarity on the thematic structure of the literature (i.e., how research efforts can be organized into major thematic areas and patterns of connections between thematic areas), which is crucial for strategic integration of the existing research. For instance, are the attention and perception mechanisms studied in cognitive psychology relevant for processing health threats (e.g., cancer, HIV/AIDS) and social threats (e.g., rejection, outgroup threat), which would support the development of an integrative model of threat processing? In recent years, researchers within specific subfields have recognized the lack of integration in the psychological research on threat and began developing integrative theoretical models of threat at the subfield level based on qualitative reviews [7–9]. As future integration efforts potentially move toward the cross-field level, clear mapping of the research landscape is essential for researchers to strategically advance integration by leveraging existing connections and addressing overlooked ones.

In this paper, we present a cross-field synthesis of the extensive psychological literature on threat to address these gaps.

### Topic modeling‌‌

Given the volume of the existing psychological literature on threat with over 51,000 relevant articles published over the past six decades, a manual review of the literature summarizing key trends and topics using human raters is not feasible. We leverage recent advances in computational text analysis, specifically topic modeling – an unsupervised machine learning approach that identifies latent topics by detecting patterns of word co-occurrence across a corpus of text documents (e.g., essays, journal abstracts, or news articles) [10,11].

#### Strengths of topic modeling.

In recent years, researchers within psychology [12,13] as well as other disciplines [14,15] have utilized topic modeling to review academic literature. These existing reviews demonstrate three key strengths of applying topic modeling to synthesize large volumes of academic research that are pertinent to the present study.

Firstly, topic modeling facilitates the identification of research gaps by revealing areas of concentrated scholarly attention, enabling researchers to identify underexplored or absent topics. To illustrate, Sperandeo et al. conducted topic modeling on the abstracts of 7,572 articles about personality and mental health, and identified research gaps through close examination of the emergent topics and themes [13]. For instance, topics on biological underpinnings of personality disorders showed a narrow focus on impulsivity, violence, and psychotic-spectrum personality disorders, leading the authors to call for broader, more integrative research on the biological, temperamental, and genetic foundations of personality pathology [13].

Secondly, topic modeling aids in mapping the thematic structure of the literature. Patterns of topic co-occurrences within documents reveal clusters of related topics with shared research focuses that form broader thematic areas within the literature. To illustrate, Healy et al. applied structural topic modeling [16] on the abstracts of 7,591 journal articles from the employability literature, identifying two main research domains from the correlation network of topics [17]. Topics within the same domain were connected but no cross-domain connections emerged, suggesting a relatively diffuse conceptual structure and a need for more integrative efforts [17].

Finally, topic modeling offers insight into how research priorities within the literature have shifted over time. Topic prevalence is estimated at the document level, reflecting the degree to which each topic contributes to a given document. By combining these estimates with document-level metadata like publication year, researchers can trace topic prevalence trends to identify topics that have grown or declined in prominence (see [16] for technical details). Temporal trends reveal the historical evolution and present state of the literature, informing research directions to leverage opportunities from emerging areas or critically re-engage with declining areas. To illustrate, Healy et al. utilized publication year as a covariate in structural topic modeling to uncover how the employability literature has shifted from focusing on workforce exclusion during much of the 20^th^ century to studying employability as an educational outcome since the 1990s [17].

### Present study

In the present study, we review psychological literature on threat over the past six decades to address three research questions. Firstly, what are the key topics that have attracted strong research interest within existing psychological literature on threat? Secondly, what do patterns of topic co-occurrences reveal about the thematic structure of the literature? Thirdly, given that topics may wax and wane in popularity, how has the prevalence of topics within the research landscape changed over time? We address these questions by analyzing 51,903 publications gathered from three scholarly databases. Specifically, we conducted structural topic modeling on the publications’ titles and abstracts, which should provide a concise and accurate representation of the key topics examined in each publication.

In addressing these questions, we leverage the three strengths of utilizing topic modeling to review academic literature highlighted above. Firstly, by critically assessing the key topics identified from the psychological literature on threat, we seek to uncover areas of inquiry that are potentially underexplored. Secondly, we examine patterns of topic co-occurrences in publications to provide insights on the thematic structure of the literature and highlight opportunities for future research to leverage existing connections and bridge observed gaps. Thirdly, through analyzing temporal trends in topic prevalence, we map shifts in the research landscape over the past decades to provide insight into the historical evolution of the landscape and its current state. In sum, this study takes stock of existing psychological research on threat to offer critical reflections on how researchers can advance the understanding of the psychology of threat by addressing gaps in current inquiries and fostering greater integration across areas of inquiry.

## Method

### Data collection

We gathered psychological literature on threat using three databases (APA PsycInfo, Scopus, Web of Science Core Collection) on 31 July 2024. APA PsycInfo is a database produced by the American Psychological Association (APA) that contains over 5 million indexed records from psychological literature and related disciplines [18]. While APA PsycInfo provides extensive coverage of psychology journals, some journals are not included in its indexing, such as regional and emerging journals as well as transdisciplinary journals that also publish psychological research. Examples of psychology journals not indexed in APA PsycInfo but indexed in Scopus and/or Web of Science Core Collection include *Cyberpsychology*, *International Journal of Educational Psychology*, and *Mediterranean Journal of Clinical Psychology*. As such, we supplemented our APA PsycInfo literature search with separate searches in Elsevier’s Scopus and Clarivate’s Web of Science Core Collection, comprehensive databases that index over 94 million [19] and over 92 million [20] records respectively. To ensure that only psychological literature was retrieved from the two broad, multidisciplinary databases, we restricted our search results in Scopus and Web of Science to the Psychology category. The search parameters used for each database and the number of records found are shown in Table 1.

**Table 1:** Search parameters and number of results by database.

	APA PsycInfo	Scopus	Web of Science
Search parameters	“threat” in title OR abstract OR keywords Filters applied: 1. Language: English 2. Publication date: up to 2023 3. Population group: Human	“threat” in Article title, abstract, keywords Filters applied: 1. Language: English 2. Publication date: up to 2023 3. Subject area: Psychology	“threat” in Topic (i.e., title, abstract, keyword plus, author keywords) Filters applied: 1. Language: English 2. Publication date: up to 2023 3. Web of Science categories: Psychology, Psychology Applied, Psychology Biological, Psychology Clinical, Psychology Developmental, Psychology Educational, Psychology Experimental, Psychology Mathematical, Psychology Multidisciplinary, Psychology Psychoanalysis
Number of records	49,369	23,681	24,292

We searched for “threat” in the title, keywords, and abstract to extract threat-related articles. Across the three databases, we filtered search results to English-language studies only to ensure linguistic consistency as topic modeling performs best on mono-lingual text data; variations in grammar, syntax, and vocabulary across languages can negatively impact model accuracy and coherence. Additionally, we set 2023 as the cut-off date for our search results to ensure the inclusion of only complete years of data, as we planned to estimate topic prevalence on a yearly basis. As our primary focus was on reviewing psychological research on threat conducted in human populations, we filtered our APA PsycInfo search results to include only results with “Human” as the population group. However, as no equivalent filter was available in Scopus and Web of Science, search results from these databases included a small set of psychological studies on threat that utilized animal models. Overall, gathering records from three databases provided comprehensive coverage of the existing psychological literature on threat and good representation of research on threat from disciplines relevant to psychological science.

### Data selection

**Fig 1:**
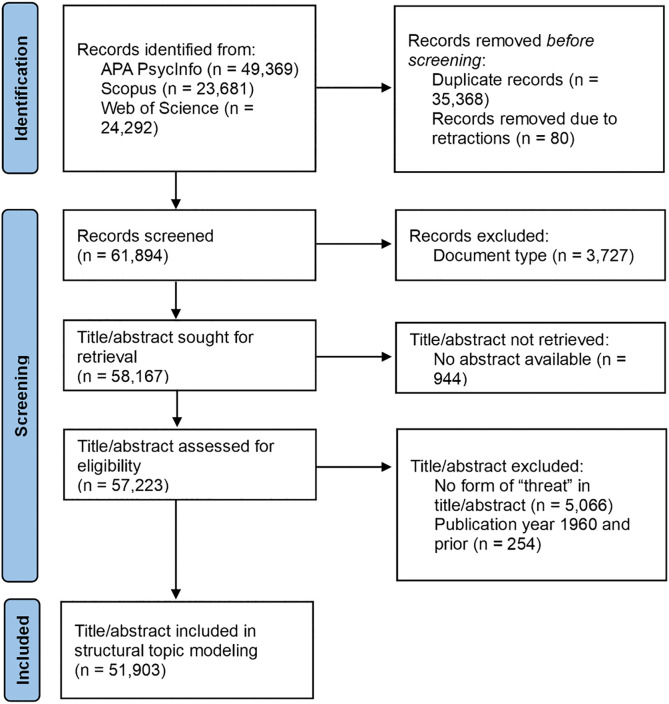
Adapted PRISMA flow diagram of record selection for structural topic modeling.

We summarized the selection of records for structural topic modeling by adapting the Preferred Reporting Items for Systematic reviews and Meta-Analyses (PRISMA) flow diagram [21] (see Fig 1). Firstly, given that some records were indexed in multiple databases, we searched for and removed duplicate records based on record information such as Digital Object Identifiers (DOIs) and titles. We also removed records with DOIs found in the Retraction Watch database [22] and records that contained retraction notices. Secondly, to focus on empirical studies, we excluded records based on database-provided document type metadata (e.g., book reviews, comments, corrections, editorials; see S1 Table for complete list). Thirdly, we excluded records with no abstract available as we used the title and abstract of each record for structural topic modeling. Finally, we removed records where “threat” (or any of its forms, e.g., “threats”, “threatened”, “non-threatening”) appeared only in the keywords and not in the title or abstract. Such records often lacked substantial discussion of threat in the main text, making them less relevant to our analysis. We also removed records published in 1960 and earlier due to data sparsity in these years, with some years not having any records and the others having very few records (i.e., 1–25). Given that one of the study goals was to estimate changes in topic prevalence over time through the structural topic model, this sparsity could lead to overfitting and poor estimates of topic prevalence trends. The final dataset contained title and abstract pairs from 51,903 records.

#### Exponential growth in threat-related records over time.

**Fig 2:**
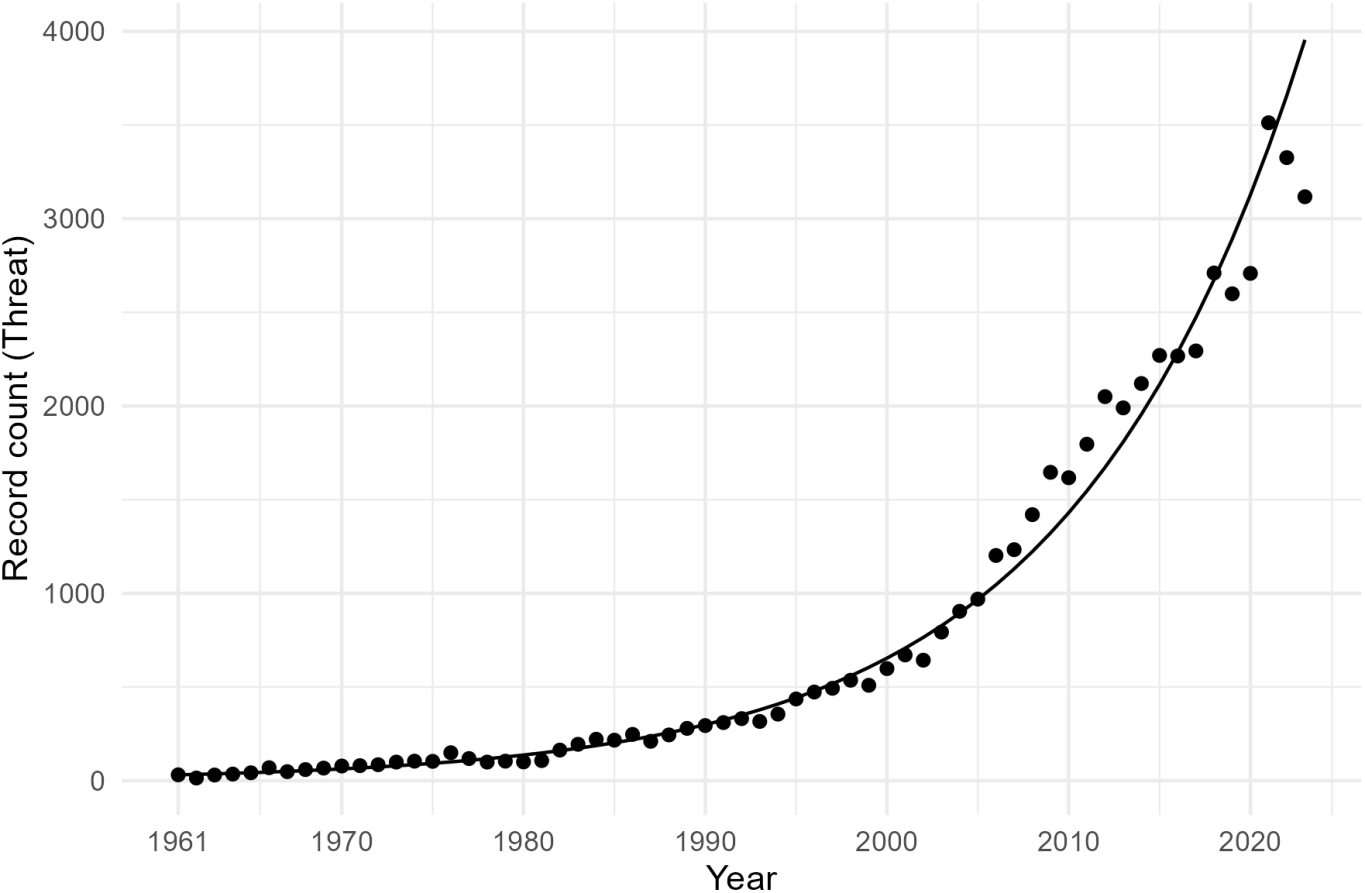
Growth trajectory of threat-related records over time.

**Fig 3:**
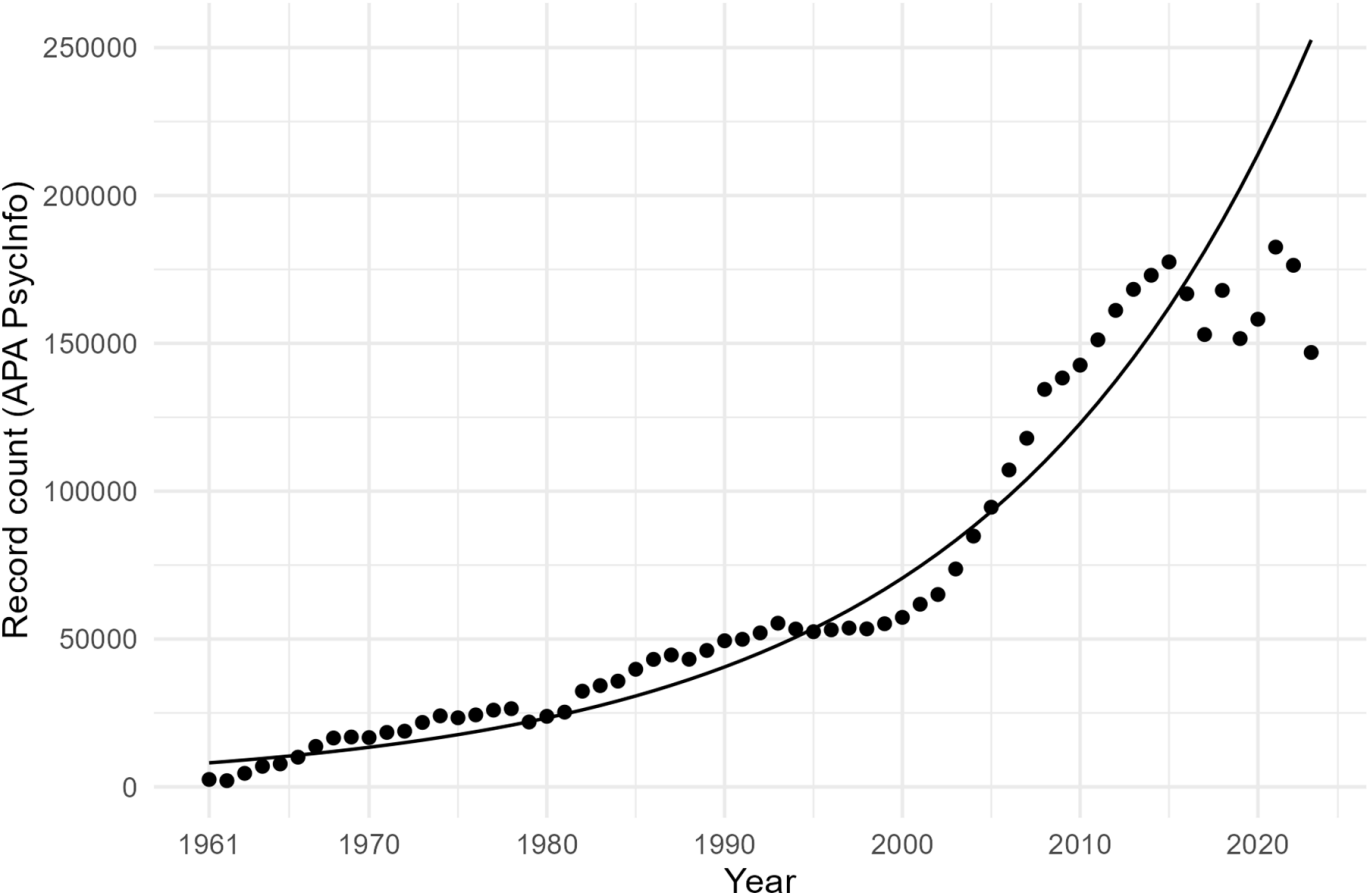
Growth trajectory of APA PsycInfo records over time.

Fig 2 shows the growth trajectory of the 51,903 records in the final dataset over time, with a log-linear regression model indicating exponential growth at 8.14% annually (adjusted *R*^2^ = .983). We assessed whether the growth in threat-related records reflected general growth in psychological literature by computing the growth trajectory of all records in APA PsycInfo over the same period (Fig 3). APA PsycInfo records also showed exponential growth, though at a lower rate of 5.70% annually (adjusted *R*^2^ = .906) and with notable deviations beginning around 2015. Threat-related records grew disproportionately faster and more consistently than psychological publications, as reflected by the higher growth rate and better model fit.

### Data preparation

To prepare the data for structural topic modeling, we combined the title and abstract of each record to form one document per record. Subsequently, we pre-processed the data to remove irrelevant information and reduce noise. Firstly, we removed metadata such as copyright notices, correction notices, and reprint statements, which were erroneously included as part of some abstracts in the databases. Next, we applied standard text preprocessing steps to the documents (see S2 Table for full preprocessing steps). Finally, we removed highly frequent and highly infrequent words from the documents to enhance topic differentiation and reduce noise in the structural topic model. Through examining the distribution of word frequency across documents, we removed five words present in at least 30% of documents (i.e., threat, study, use, result, research), which were common academic writing terms unlikely to contribute to identifying distinct topics. We also removed 107,839 words that appeared in 0.25% or fewer documents, as Monte Carlo experiments [23] showed that a pruning threshold of 0.25% to 0.5% provided the most improvement for topic quality. Given that word usage across documents in our corpus was quite diverse as evidenced by only five words appearing in at least 30% of documents, we chose the more conservative threshold of 0.25% for pruning.

### Structural topic modeling

Using the *stm* package [24] in R, we fitted a structural topic model on our corpus of 51,903 documents. Publication year was included as a nonlinear covariate of topic prevalence to estimate its variation over time.

Following established procedures for topic model selection (see [16,25] for further information), we first generated candidate models ranging from five to 100 topics in intervals of five. Based on quantitative metrics of model fit and topic quality, we identified 20–30 topics as the optimal range for further evaluation. Subsequently, we generated candidate models from 20 to 30 topics in intervals of one and shortlisted the 24-, 25-, and 26-topic models, which showed the best balance between model fit and topic quality. For the three shortlisted models, we generated an additional set of quantitative metrics to conduct a finer-grained assessment of topic quality and qualitatively evaluated the interpretability of the topics within the three models by reviewing the top words for each topic. We selected 25 topics, which showed better topic quality and higher interpretability, as the optimal topic number (see S1 File for details on topic model selection and model evaluation).

### Analyses to address research questions

To address the first research question regarding key topics within the psychological literature on threat, we reviewed the top words and documents associated with each of the 25 topics to create descriptive labels that captured the conceptual content of each topic. Additionally, we collated the most frequent APA classification codes assigned to documents with the highest topic prevalence (top 5^th^ percentile) for each topic to gain insight on the disciplinary subfield in which each topic was primarily situated. APA classification codes are assigned by human indexers to all records indexed in APA PsycInfo based on the record’s primary subject matter [26]. These codes were available for 45,608 (87.87%) of the documents in our dataset indexed in APA PsycInfo. Given the lack of a standardized method for assigning disciplinary subfields to documents or journals, this approach provided the most systematic and feasible method for understanding the disciplinary orientation of each topic.

To address the second research question on the thematic structure of the literature, we conducted exploratory graph analysis (EGA) [27] on the prevalence of the 25 topics across documents to map a network of connections between topics based on co-occurrence patterns. Specifically, we used the *EGAnet* package in R [28] to conduct a bootstrapped EGA procedure with 1,000 iterations, which allowed us to assess the stability of the network structure and identify clusters of topics through community detection. We also analyzed the topic co-occurrence network to assess overall network organization, cluster connectivity patterns, and structurally important topics (i.e., hubs and bridges) within the network.

To address the third research question on temporal trends of topic prevalence within the literature, we conducted two sets of analyses. Firstly, we ran a Beta regression separately for each topic with publication year as the predictor. We utilized the *stmprevalence* package in R [29], which improves the robustness of the regression estimates by accounting for the uncertainty inherent in topic modeling. However, this approach is limited to descriptive analyses of the topic prevalence trends. Hence, we also fitted a generalized additive model to the temporal trend in prevalence for each topic and estimated the first derivative using the *gratia* package in R [30], which allowed us to identify the time periods with significant changes in topic prevalence.

## Results and discussion

### Topics identified through structural topic modeling

Table 2 shows the 25 topics from the structural topic model, organized by the four clusters identified from EGA and their corresponding labels. For each topic, Table 2 presents a brief description of the topic content, top 10 words that were frequent in and exclusive to the topic (FREX), average prevalence across all documents, and top three APA classification code(s) that occurred most frequently among documents with the highest topic prevalence (top 5^th^ percentile). Cluster and topic labels represent our interpretive assessment of predominant thematic content and should be considered provisional rather than definitive designations.

**Table 2:** Topics from the structural topic model organized by EGA clusters.

Topic	Topic label	Description	Average prevalence	Top 10 FREX words	Top three APA classification codes
***Cluster 1 (Threat processing mechanisms)***					
P1	Neural threat processing in anxiety	• Neural activation patterns associated with anxiety • Focus on brain structures (e.g., amygdala, prefrontal cortex)	0.038	anxiety, amygdala, anxious, word, cortex, neural, functional, brain, prefrontal, activation	Psychological & Physical Disorders (47.62%) Physiological Psychology & Neuroscience (29.82%) Health & Mental Health Treatment & Prevention (12.03%)
P2	Conditioned/reflexive physiological responses	• Learned and reflexive physiological reactions to threat (e.g., fear conditioning, startle) • Human participants and animal models	0.042	animal, extinction, startle, shock, conditioning, aversive, predator, signal, defensive, generalization	Physiological Psychology & Neuroscience (45.20%) Human Experimental Psychology (20.38%) Psychological & Physical Disorders (17.82%)
P3	Processing of facial threat cues	• Perception and evaluation of facial expressions linked with threat (e.g., fear, anger) • Focus on attentional orientation	0.036	facial, angry, neutral, visual, fearful, expression, attention, face, eye, picture	Human Experimental Psychology (31.43%) Psychological & Physical Disorders (26.77%) Physiological Psychology & Neuroscience (23.23%)
P4	Cognitive processing in psychopathology	• Cognitive processes (e.g., beliefs, rumination) that shape threat interpretation and response • Focus on psychological disorders (e.g., paranoia, psychosis)	0.020	personality, behaviour, shame, worry, cognition, cognitive, loneliness, style, thought, trait	Psychological & Physical Disorders (41.87%) Health & Mental Health Treatment & Prevention (18.23%) Personality Psychology (16.26%)
P5	Motivational and affective processing	• Motivational tendencies and emotional reactivity (e.g., reward sensitivity, disgust)	0.027	emotion, avoidance, affective, anger, sensitivity, disgust, fear, negative, implicit, reward	Psychological & Physical Disorders (22.84%) Physiological Psychology & Neuroscience (14.87%) Personality Psychology (14.06%)
P6	Physiological stress responses	• Threat and challenge appraisals of stressors • Physiological reactivity to stress (e.g., salivary cortisol, cardiovascular activation)	0.030	pain, stressor, stress, appraisal, cortisol, physiological, heart, cardiovascular, stressful, reactivity	Physiological Psychology & Neuroscience (31.38%) Psychological & Physical Disorders (22.08%) Health & Mental Health Treatment & Prevention (13.59%)
***Cluster 2 (Health and clinical threats)***					
H1	Illness threat and coping	• Threat appraisals of illness among individuals with serious health conditions (e.g., cancer, dementia) and their caregivers • Impact on coping strategies and psychosocial adjustment	0.032	cancer, cop^a^, well_being, distress, resilience, sleep, illness, breast, psychological, life	Psychological & Physical Disorders (40.17%) Health & Mental Health Treatment & Prevention (32.88%) Personality Psychology (6.98%)
H2	Role of threat perceptions in psychopathology assessment	• Development and validation of assessment tools for psychological disorders, with perceived threat as a measure • Role of perceived threat in distinguishing symptom profiles within disorders	0.048	ptsd, scale, symptom, posttraumatic, depression, depressive, trauma, score, traumatic, item	Psychological & Physical Disorders (59.75%) Psychometrics & Statistics & Methodology (20.34%) Health & Mental Health Treatment & Prevention (18.01%)
H3	Children/adolescents’ threat experiences in family contexts	• Young individuals’ experiences of threat in family environments, particularly interparental conflict • Impact on developmental outcomes and well-being	0.027	child, parent, mother, adolescent, parental, childhood, youth, adolescence, family, adversity	Psychological & Physical Disorders (33.67%) Developmental Psychology (25.72%) Health & Mental Health Treatment & Prevention (21.03%)
H4	Intimate partner violence	• Coercive control through physical, sexual, and firearm-related violence in abusive relationships • Impact on victims’ safety, mental health, and help-seeking behaviors	0.024	victim, ipv, assault, violence, victimization, abuse, sexual, rape, perpetrator, harassment	Psychological & Physical Disorders (58.58%) Health & Mental Health Treatment & Prevention (16.98%) Forensic Psychology & Legal Issues (7.38%)
H5	Self/other harm in clinical contexts	• Patients presenting with threats of self-harm or harm to others • Clinical management and risk assessment in psychiatric and medical settings	0.036	suicide, nurse, suicidal, hospital, therapy, treatment, client, patient, physician, psychiatric	Health & Mental Health Treatment & Prevention (56.59%) Psychological & Physical Disorders (27.54%) Health & Mental Health Personnel Issues (19.72%)
H6	Risky health behaviors	• Physical health threats arising from substance use (e.g., alcohol, drugs) and infectious diseases (e.g., HIV, hepatitis C) • Focus on behaviorally mediated risks	0.041	hiv, risk, drug, old, aid, alcohol, driver, respondent, adult, age	Psychological & Physical Disorders (37.87%) Health & Mental Health Treatment & Prevention (28.81%) Developmental Psychology (9.79%)
H7	Pandemic threat	• Large-scale health threats stemming from infectious disease outbreaks (e.g., Ebola, COVID-19)	0.024	covid_19, pandemic, climate, chinese, 2020, vaccine, uncertainty, covid, vaccination, china	Health & Mental Health Treatment & Prevention (28.89%) Psychological & Physical Disorders (24.40%) Social Processes & Social Issues (15.52%)
***Cluster 3 (Social psychological threats)***					
S1	Stereotype threat in academic contexts	• Impact of negative stereotypes about social group membership on performance • Focus on academic testing and achievement settings	0.033	stereotype, performance, college, achievement, math, academic, student, university, feedback, test	Educational & School Psychology (39.33%) Social Psychology (17.02%) Human Experimental Psychology (8.45%)
S2	Gendered interpersonal responses	• Interpersonal responses to threat (e.g., jealousy, aggression) • Gender differences arising from gender identity, norms, and roles	0.030	attachment, men, male, romantic, masculinity, couple, narcissism, aggressive, jealousy, aggression	Social Psychology (25.65%) Social Processes & Social Issues (23.52%) Psychological & Physical Disorders (17.47%)
S3	Self and social identity threat	• Threats to personal self-concept (e.g., self-esteem, status) • Threats to social identity (e.g., group membership, in-group status)	0.044	social, self_esteem, exclusion, collective, ingroup, rejection, identity, group, member, ostracism	Social Psychology (50.52%) Social Processes & Social Issues (11.43%) Personality Psychology (11.08%)
S4	Attitudinal and behavioral responses to threat communications	• Threats conveyed through messages, social interactions or situational cues • Impact on attitudes and behaviors, particularly compliance and reactance	0.062	message, intention, motivation, efficacy, choice, decision, preference, appeal, judgment, reactance	Social Psychology (21.31%) Health & Mental Health Treatment & Prevention (18.78%) Personality Psychology (10.32%)
***Cluster 4 (Collective threats)***					
C1	Security threat in educational institutions	• Threats to school safety and functioning (e.g., school shootings, campus violence) • Focus on threat assessment practices, law enforcement responses, institutional policy	0.028	police, law, officer, teacher, enforcement, school, justice, criminal, court, prison	Educational & School Psychology (34.77%) Forensic Psychology & Legal Issues (30.72%) Psychological & Physical Disorders (16.16%)
C2	National security threat	• Threats to nations’ security and sovereignty (e.g., terrorism, weapons of mass destruction) • Situated in broader regional or international dynamics	0.060	terrorism, war, terrorist, nuclear, international, book, military, society, century, politics	Social Processes & Social Issues (51.47%) Psychological & Physical Disorders (11.45%) Health & Mental Health Treatment & Prevention (9.20%)
C3	Organizational threat	• External and internal threats to organizations’ competitive advantage, market performance or long-term viability	0.042	employee, organizational, firm, market, consumer, job, organization, manager, product, leadership	Organizational Psychology & Human Resources (62.08%) Consumer Psychology (13.42%) Social Processes & Social Issues (5.68%)
C4	Cybersecurity threat	• Threats to digital systems, data infrastructure, and networks of organizations, institutions, and societies • Technological and strategic approaches for threat detection, mitigation, and prevention	0.066	technology, security, privacy, application, user, internet, tool, digital, systematic, review	Psychometrics & Statistics & Methodology (16.09%) Organizational Psychology & Human Resources (14.89%) Communication Systems (12.41%)
C5	Public health institutional threat	• Role of healthcare institutions in addressing threats to population health • Systemic vulnerabilities that threaten institutional efficacy	0.042	service, healthcare, health, barrier, program, obesity, intervention, disaster, provider, emergency	Health & Mental Health Treatment & Prevention (60.38%) Psychological & Physical Disorders (16.09%) Social Processes & Social Issues (7.02%)
C6	Discursive analysis of threat experiences	• Threat experiences of marginalized, minority, and situationally vulnerable groups • Qualitative discourse analysis of how experiences are constructed and given meaning	0.047	theme, narrative, qualitative, discourse, language, construction, interview, space, in_depth, culture	Health & Mental Health Treatment & Prevention (20.53%) Psychological & Physical Disorders (12.33%) Educational & School Psychology (9.51%)
C7	Intergroup threat	• Realistic (e.g., competition for resources, physical safety) and symbolic (e.g., cultural values, traditions) threats posed by outgroups to one’s ingroup • Impact on group members’ attitudes and behaviors towards outgroups	0.036	immigrant, racial, ethnic, prejudice, white, black, minority, immigration, american, race	Social Processes & Social Issues (49.60%) Social Psychology (24.93%) Health & Mental Health Treatment & Prevention (6.21%)
C8	Existential threat	• Threats about fundamental existential concerns (e.g., mortality, meaning) • Focus on psychoanalytic and philosophical approaches	0.084	chapter, concept, dream, meaning, death, author, defense, mind, example, describe	Health & Mental Health Treatment & Prevention (27.17%) Personality Psychology (18.13%) Psychological & Physical Disorders (17.20%)

^a^The NLTK WordNet lemmatizer incorrectly reduced “coping” to “cop” in 6,303 of the 8,057 occurrences (78.23%) of “coping”, with the remaining 21.77% retained as “coping”. When we inspected the terms lemmatized as “cop” (n = 6,450), 97.72% originated from “coping,” indicating that the “cop” term in the lemmatized data generally represented instances of “coping”.

The 25 topics revealed considerable diversity in existing threat investigations. Research spanned diverse populations (e.g., clinical patients, children/adolescents, marginalized groups) and addressed threats across varied application domains (e.g., romantic relationships, public health, cybersecurity). Top-occurring APA classification codes exhibited disciplinary breadth, with topics situated in clinical psychology (H1: Psychological & Physical Disorders), cognitive psychology (P3: Human Experimental Psychology), social psychology (S3: Social Psychology), and organizational psychology (C3: Organizational Psychology & Human Resources).

Notably, only a subset of topics (six topics in Cluster 1) focused on fundamental psychological mechanisms underlying threat processing and response such as attentional biases, emotional regulation, and physiological reactivity. In contrast, most topics were organized around a shared focus on specific threat types, including infectious diseases, intergroup conflict, and negative stereotypes. The dominance of topics focused on specific threat types indicates that psychological research on threat is primarily structured around particular threat domains rather than psychological mechanisms, consistent with Jonas et al.‘s observation that social psychological research tends to focus on specific threat types [7] and suggesting that this tendency extends to the broader psychological literature.

#### Thematic areas.

The topic co-occurrence network exhibited small-world properties (*σ* = 1.48) as reflected in both strong local clustering (*γ* = 1.45) and short connection pathways (*λ* = 0.98), indicating that topics were organized into distinct groups while maintaining efficient connections across the broader research landscape.

**Fig 4:**
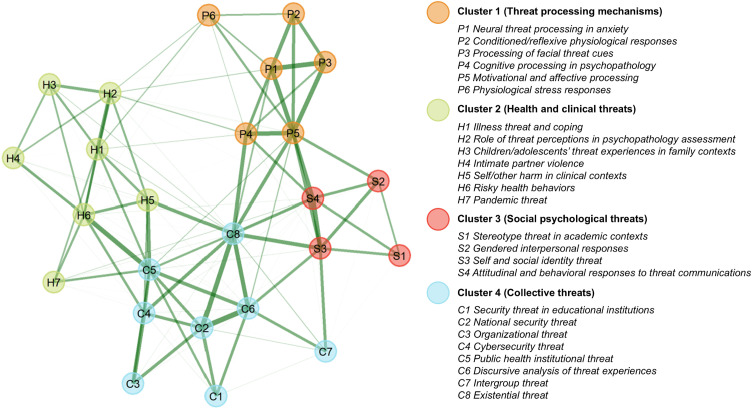
Network structure of topic co-occurrences.

The bootstrapped EGA identified a four-cluster solution as the most frequently occurring (75.80%) network structure across 1,000 iterations. Fig 4 shows the four-cluster network structure, with node colors representing the clusters identified through community detection and line thickness representing the strength of connection between nodes. The network structure showed high stability, with 23 out of 25 topics exhibiting replication proportions above 0.75 [31] (see S4 Table for cluster assignments of each topic across bootstrapped iterations).

The four clusters revealed the key research domains within psychological literature on threat. Examination of cluster composition reflected organization primarily along disciplinary boundaries and levels of social organization. The first domain (Cluster 1) comprised work on psychological mechanisms underlying threat perception and response, situated within clinical, cognitive, and biological psychology. The other three domains examined threat types ranging from individual to collective levels. Cluster 2 focused on threats related to individuals’ physical health and psychological well-being, anchored in clinical psychology. Cluster 3 focused on threats related to individuals’ social identity and interpersonal contexts, anchored in social psychology. Cluster 4 primarily focused on threats experienced by collectives including educational institutions, organizations, and nations, with diverse disciplinary associations reflecting the breadth of collective settings studied.

*C8 Existential threat* exhibited the lowest replication proportion among the 25 topics (0.695), indicating relatively unstable cluster assignment across bootstrapped iterations. Nevertheless, inter-topic connections showed that *C8 Existential threat*’s strongest connections were with Cluster 4 topics (*C2 National security threat*, *C6 Discursive analysis of threat experiences*), consistent with the modal assignment of *C8 Existential threat* to Cluster 4. At a conceptual level, research on existential threat intersects with collective threat topics, as concerns about existence and survival operate at both individual and collective levels. For instance, uncertainty, lack of control and lack of meaning are often experienced within collective contexts that highlight these issues for individuals (e.g., via lack of institutional structure, collective deprivation or threat).

While the emergence of research domains with clear disciplinary associations indicated potential disciplinary siloing, the extent of connectivity within and between domains remained unclear.

### Cluster connectivity

We examined network connectivity patterns through multidimensional scaling (MDS) and network analysis of edge strengths, structural hubs, and cross-cluster bridges.

#### Multidimensional scaling.

**Fig 5:**
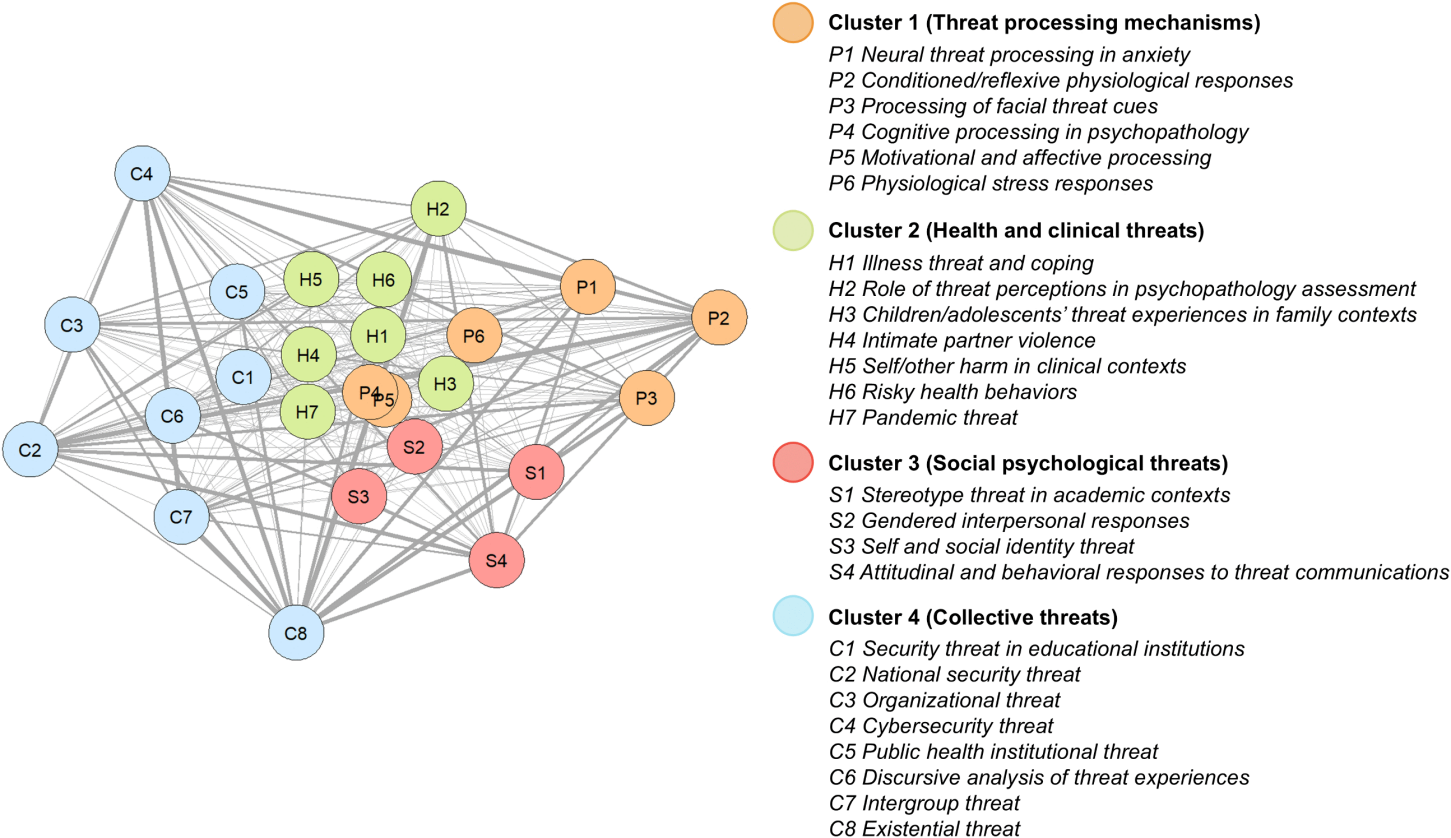
Two-dimensional MDS plot of topic relationships (EBICglasso network).

We conducted MDS on the 25 topics to visualize the structural relationships between topics in a two-dimensional space (Fig 5). Nodes represent topics, colored based on cluster membership identified through EGA, with edge thickness reflecting regularized partial correlation strength from the EBICglasso network. The MDS layout showed moderate stress (0.268), typical for complex networks reduced to two dimensions. The underlying EBICglasso network demonstrated high stability (edge stability = 0.750).

The MDS revealed spatial separation by research domain, with topics from the same cluster located closer together than topics from other clusters. Clusters varied in spatial compactness, with Cluster 2 showing the tightest spatial clustering and Cluster 4 the greatest spatial dispersion. Further, the MDS showed spatial organization by level of analysis, with collective threats on the left and individual-focused research on the right.

#### Network structure.

**Fig 6:**
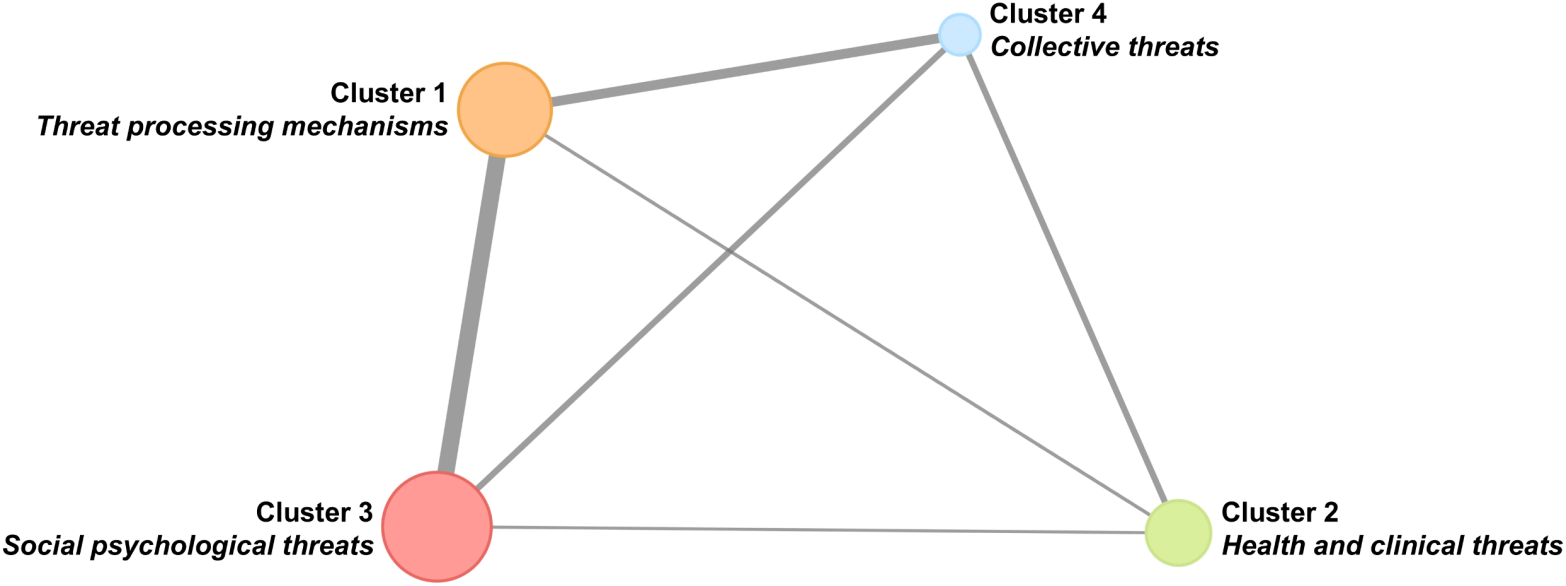
Intra- and inter-cluster connectivity.

Fig 6 shows the degree of internal cohesion and external connectivity averaged across observed connections for the four thematic clusters, with larger nodes indicating stronger within-cluster connections and thicker lines indicating stronger between-cluster connections (see Table 3 for average intra- and inter-cluster edge strengths). Cluster 3 showed the highest internal cohesion. Consistent with the spatial dispersion observed in the MDS, Cluster 4 had the lowest internal cohesion. Regarding cross-cluster connectivity, Cluster 1 showed strong connectivity with Clusters 3 and 4 but weak connectivity with Cluster 2. Cluster 2 also showed weak connectivity with Cluster 3.

**Table 3:** Intra- and inter-cluster connectivity.

Cluster	Average intra-cluster edge strength	Average inter-cluster edge strength		
		Cluster 1	Cluster 2	Cluster 3
Cluster 1 (Threat processing mechanisms)	0.475	–		
Cluster 2 (Health and clinical threats)	0.368	0.030	–	
Cluster 3 (Social psychological threats)	0.568	0.209	0.024	–
Cluster 4 (Collective threats)	0.340	0.112	0.066	0.065

We identified structural hubs through four centrality measures, namely strength centrality (total connectivity based on co-occurrence patterns), betweenness centrality (topics that lie on pathways between other topics), closeness centrality (efficiency of reaching all other topics), and eigenvector centrality (connection to other highly connected topics). *C8 Existential threat*, *P5 Motivational and affective processing*, and *C5 Public health institutional threat* demonstrated the highest centrality across the four measures, indicating that these topics function as structural hubs that mediate information flow and connect otherwise distant topics in the network (see S5 Table for topics’ centrality scores).

Analysis of cross-cluster connections revealed 16 topic pairs with connection weights higher than one standard deviation above the mean (weight > 0.171). We characterized the content of these cross-cluster bridges to understand conceptual overlaps between thematic areas by examining shared APA PsycInfo descriptors, which are staff-assigned terms that characterize the subject matter of APA PsycInfo records using psychology-specific controlled vocabulary [32]. Available for 45,608 (87.87%) of the documents in our dataset indexed in APA PsycInfo, these descriptors provided the most systematic approach for characterizing content overlap in cross-cluster bridges. Output from the structural topic model (i.e., words most strongly associated with each topic and FREX words) was not suitable as the words were optimized for topic differentiation and showed minimal overlap between topics. For each topic pair, we identified descriptors that appeared in at least 3% of the documents with the highest topic prevalence (top 5^th^ percentile) for both topics in the pair. Table 4 presents the 16 cross-cluster topic pairs, connection weights, and shared APA descriptors for each pair.

**Table 4:** Cross-cluster bridges.

Topic	Topic	Connection weight	Shared APA PsycInfo descriptors^a^
***Bridges between Cluster 1 (Threat processing mechanisms) and Cluster 3 (Social psychological threats)***			
P5 Motivational and affective processing	S4 Attitudinal and behavioral responses to threat communications.	0.306	Fear, Motivation, Coping Behavior
P5 Motivational and affective processing	S3 Self and social identity threat.	0.269	Motivation
P5 Motivational and affective processing	S2 Gendered interpersonal responses.	0.228	Anxiety, Motivation, Coping Behavior, Stress
P4 Cognitive processing in psychopathology	S4 Attitudinal and behavioral responses to threat communications.	0.213	Coping Behavior, Fear, Models
***Bridges between Cluster 1 (Threat processing mechanisms) and Cluster 4 (Collective threats)***			
C8 Existential threat	P4 Cognitive processing in psychopathology	0.287	Anxiety, Coping Behavior, Cognitive Processes, Emotions, Stress, Fear
C8 Existential threat	P5 Motivational and affective processing	0.28	Emotions, Fear, Anxiety, Motivation, Coping Behavior, Cognitive Processes, Stress
***Bridges between Cluster 2 (Health and clinical threats) and Cluster 4 (Collective threats)***			
C5 Public health institutional threat	H6 Risky health behaviors	0.362	Public Health, Risk Factors, HIV, COVID-19, Prevention, Health, Pandemics, Health Behavior
C5 Public health institutional threat	H5 Self/other harm in clinical contexts	0.288	Mental Health, Mental Disorders, Risk Factors
C5 Public health institutional threat	H7 Pandemic threat	0.194	COVID-19, Pandemics, Public Health, Mental Health, Climate Change, Well Being, Risk Factors, Health Behavior
C5 Public health institutional threat	H1 Illness threat and coping	0.187	Mental Health, Well Being, COVID-19, Health, Pandemics, Risk Factors, Mental Disorders, Health Behavior
C8 Existential threat	H5 Self/other harm in clinical contexts	0.275	Psychotherapy, Psychotherapeutic Processes
H6 Risky health behaviors	C4 Cybersecurity threat	0.209	Risk Factors, Risk Assessment
***Bridges between Cluster 3 (Social psychological threats) and Cluster 4 (Collective threats)***			
C8 Existential threat	S3 Self and social identity threat.	0.297	Self-Concept, Motivation
C8 Existential threat	S4 Attitudinal and behavioral responses to threat communications.	0.263	Motivation, Fear, Self-Concept, Theories, Coping Behavior
S3 Self and social identity threat.	C7 Intergroup threat	0.237	Intergroup Dynamics, Ingroup Outgroup, Social Identity, Prejudice, Stereotyped Attitudes, Minority Groups, Test Construction, Conflict
S3 Self and social identity threat.	C6 Discursive analysis of threat experiences	0.215	Self-Concept

^a^“Threat” emerged as a shared descriptor for all topic pairs due to the study focus and is not included in the table for interpretive clarity. Remaining shared descriptors are ordered by average frequency across both topics in each pair.

Structural hubs featured in 12 out of 16 cross-cluster bridges. *C5 Public health institutional threat* served as the primary connector between Cluster 2 and Cluster 4, while *P5 Motivational and affective processing* served as the primary connector between Cluster 1 and Cluster 3. *C8 Existential threat* functioned as a general hub, serving as the primary connector between Cluster 1 and Cluster 4 but also bridging Cluster 4 to the other two clusters (Cluster 2, Cluster 3).

#### Synthesis of cluster connectivity findings.

Analyses of network connectivity patterns revealed limited connectivity between research domains, with cross-domain connections concentrated through specific hub topics rather than distributed broadly across the network. As researchers reflect on the level of integration within threat research and develop integrative models [8,9], these hubs provide strategic starting points for future efforts to advance theoretical synthesis across thematic areas in existing literature.

The strongest cross-cluster connectivity emerged between Cluster 1 (Threat processing mechanisms) and Cluster 3 (Social psychological threats), mediated primarily by the structural hub topic *P5 Motivational and affective processing*. This connection indicates that social psychological threat research commonly incorporates emotional and motivational processes, suggesting some integration between mechanism-focused and domain-specific research [33,34]. Separately, *C5 Public health institutional threat* mediated connectivity between Cluster 2 (Health and clinical threats) and Cluster 4 (Collective threats), underscoring the position of public health phenomena at the intersection of individual health concerns and collective institutional responses [35,36]. In contrast, Cluster 2 showed weak connectivity with both Cluster 1 and Cluster 3, evidenced by both weak between-cluster edge strengths and absence of cross-cluster bridges between these cluster pairs. This limited connectivity reveals that health and clinical threat research has developed relatively independently from mechanism-focused and social psychological approaches, indicating both a gap and an opportunity for cross-domain integration in future work. Interestingly, Cluster 2 topics occupied the center of the MDS visualization, which would have suggested central hub status in the network. However, given the edge strength and cross-cluster bridging analyses, Cluster 2’s spatial centrality paradoxically indicated isolation rather than integration, with the central position reflecting weak cross-domain connectivity rather than strong hub-like connections.

No central hub domain emerged from the network connectivity patterns. Cluster 1 (Threat processing mechanisms) would be the logical candidate, as core psychological mechanisms should underlie perception and response to the diverse threat types examined in other clusters. Yet, Cluster 1 showed substantial connectivity gaps. Besides having weak connections and no cross-cluster bridges to Cluster 2 (Health and clinical threats), Cluster 1 was spatially distant from Cluster 4 (Collective threats) in the MDS visualization despite the presence of two cross-cluster bridges mediated by *C8 Existential threat*. These patterns suggest that psychological threat processing mechanisms have not played a central integrative role across threat research domains. Though the absence of a central hub domain made cross-domain connections mediated through topic-level hubs even more critical, the primary hub facilitating these connections (*C8 Existential threat*) had uncertain foundations. While the topic showed the broadest connectivity as the primary bridge between Cluster 1 and Cluster 4 and also bridging Cluster 4 to the other two clusters, the basis for these connections was ambiguous. The broad connectivity could reflect genuine conceptual overlap – connections to Cluster 1 may stem from shared emphasis on internal psychological mechanisms, while links to Clusters 2 and 3 may reflect alignment in concerns related to psychological well-being and identity. However, the prevalence of some generic academic terms in the topic’s top FREX words (e.g., “chapter”, “example”, “describe”) raises the possibility that the connectivity may partly reflect linguistic overlap in academic writing rather than substantive conceptual integration.

Finally, within-domain cohesion (i.e., the extent to which topics within a cluster form a coherent research domain) varied across clusters, as reflected in the strength of within-cluster connections and spatial proximity in the MDS visualization. Cluster 3 (Social psychological threats) exhibited the highest cohesion, consistent with the four topics’ shared theoretical foundations in social psychology (e.g., social identity theory). In contrast, Cluster 4 (Collective threats) showed the lowest cohesion, evidenced by both weak within-cluster edge strength and greater spatial dispersion of topics in the MDS visualization. The low internal cohesion reflected the thematic heterogeneity of collective threats, which encompassed variation across levels of social organization, institutional contexts, and disciplinary subfields. These patterns suggest that Cluster 4 may not constitute a coherent research domain in which topics share theoretical frameworks and are frequently examined together; rather, the cluster appears to have emerged primarily from a shared focus on collectives.

### Temporal trends

#### Diversification in research attention.

**Fig 7:**
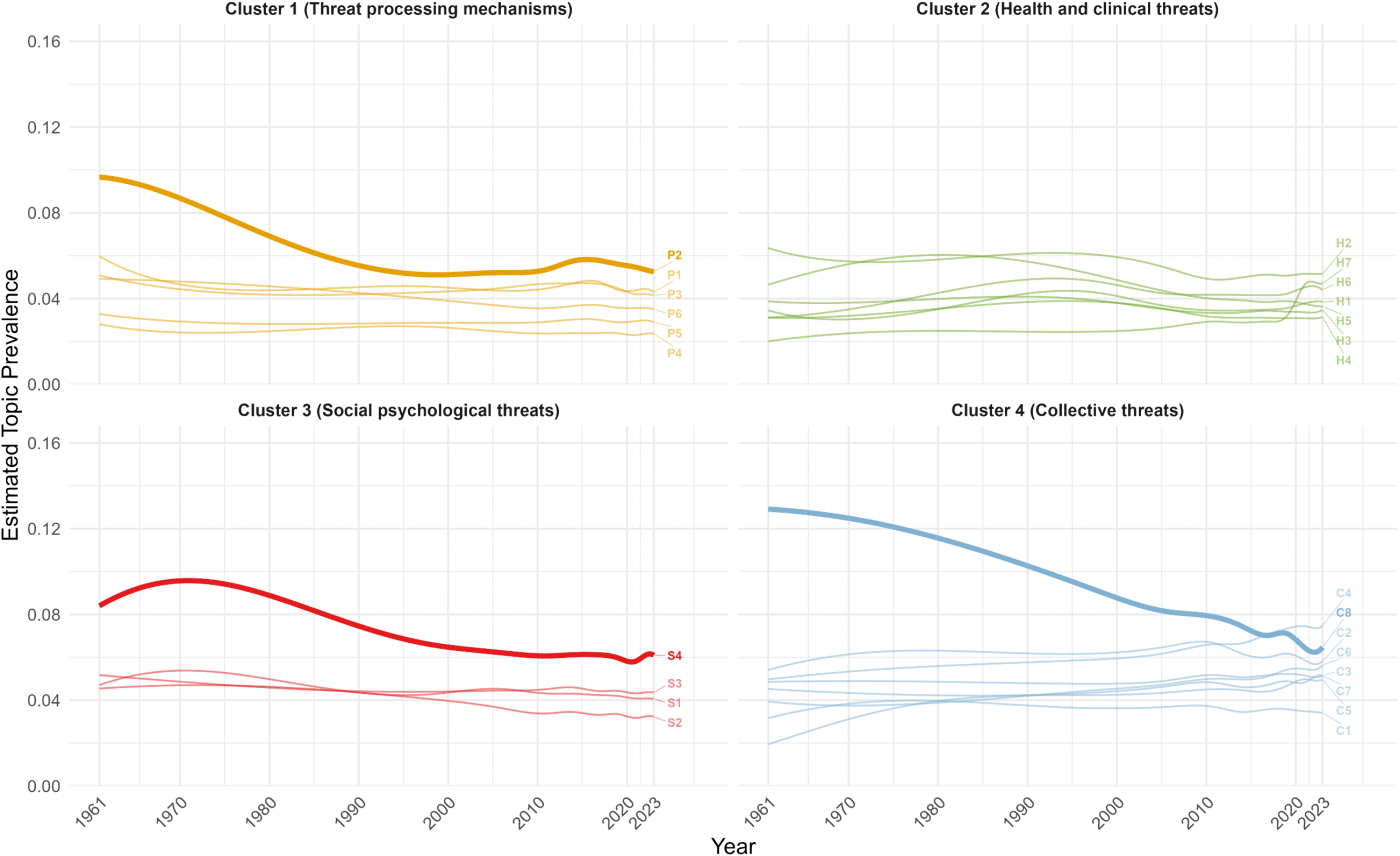
Temporal trends in topic prevalence across topics from 1961 to 2023. Topics are grouped by cluster, with labels at the end of each trendline indicating the topic number. Trendlines for the three initially dominant topics are in bold.

Fig 7 illustrates the overall temporal trends in topic prevalence across the 25 topics. Psychological literature on threat appears to have moved from an early concentration on a few dominant topics to a broader distribution of attention across a larger set of topics. In early decades, three topics were dominant. Over time, the three initially dominant topics declined in prevalence while the other topics increased in prevalence, with the topic prevalence of all topics converging around a similar prevalence range in recent decades.

#### Differential cluster-level trajectories.

**Fig 8:**
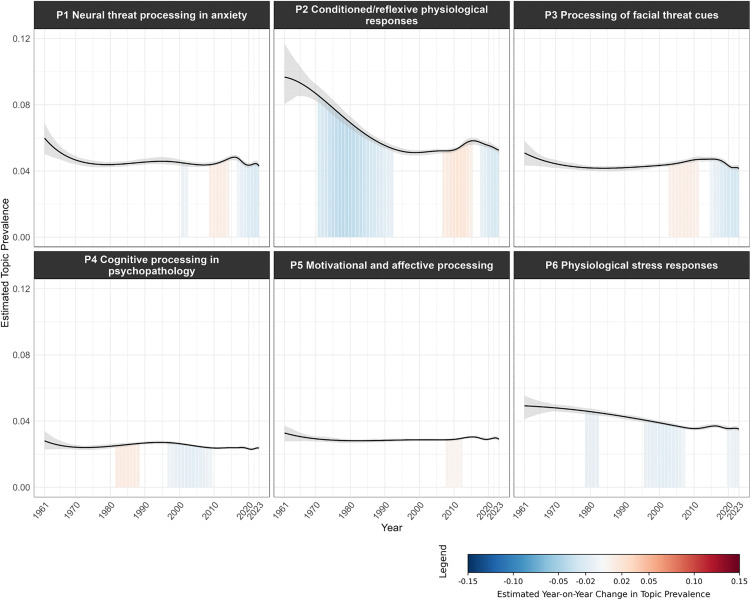
Temporal trends in topic prevalence (Cluster 1 Threat processing mechanisms). The grey band represents the 95% confidence interval of the estimated topic prevalence. Shaded regions under the curve indicate periods of statistically significant year-on-year change in topic prevalence. Blue shading denotes a significant decrease, red shading a significant increase, and unshaded areas indicate non-significant change. Color intensity reflects the magnitude of change, with darker shades representing larger changes.

**Fig 9:**
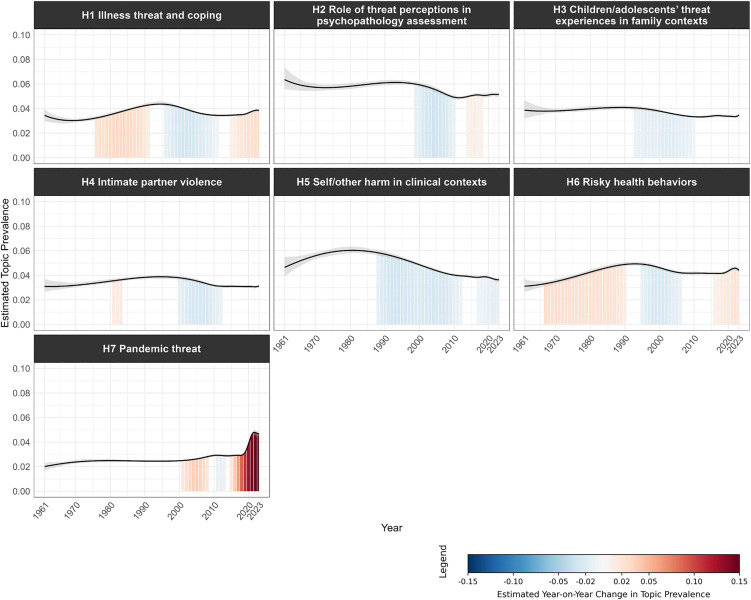
Temporal trends in topic prevalence (Cluster 2 Health and clinical threats). The grey band represents the 95% confidence interval of the estimated topic prevalence. Shaded regions under the curve indicate periods of statistically significant year-on-year change in topic prevalence. Blue shading denotes a significant decrease, red shading a significant increase, and unshaded areas indicate non-significant change. Color intensity reflects the magnitude of change, with darker shades representing larger changes.

**Fig 10:**
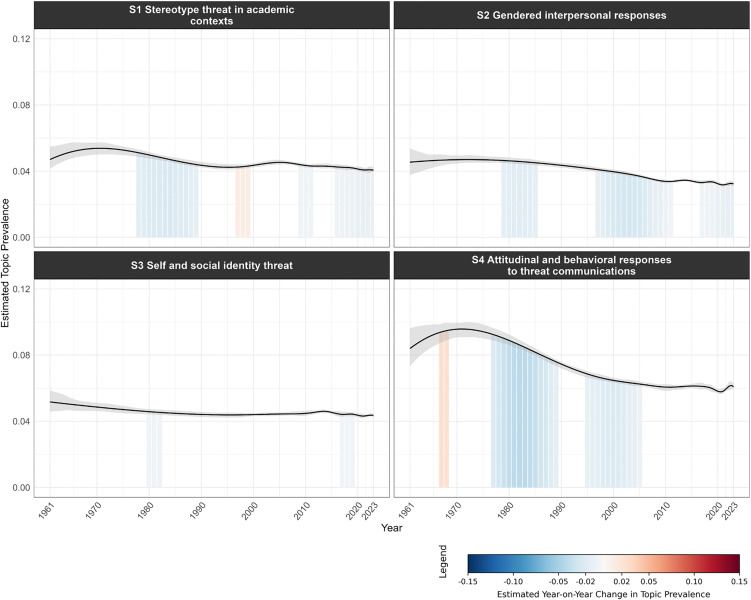
Temporal trends in topic prevalence (Cluster 3 Social psychological threats). The grey band represents the 95% confidence interval of the estimated topic prevalence. Shaded regions under the curve indicate periods of statistically significant year-on-year change in topic prevalence. Blue shading denotes a significant decrease, red shading a significant increase, and unshaded areas indicate non-significant change. Color intensity reflects the magnitude of change, with darker shades representing larger changes.

**Fig 11:**
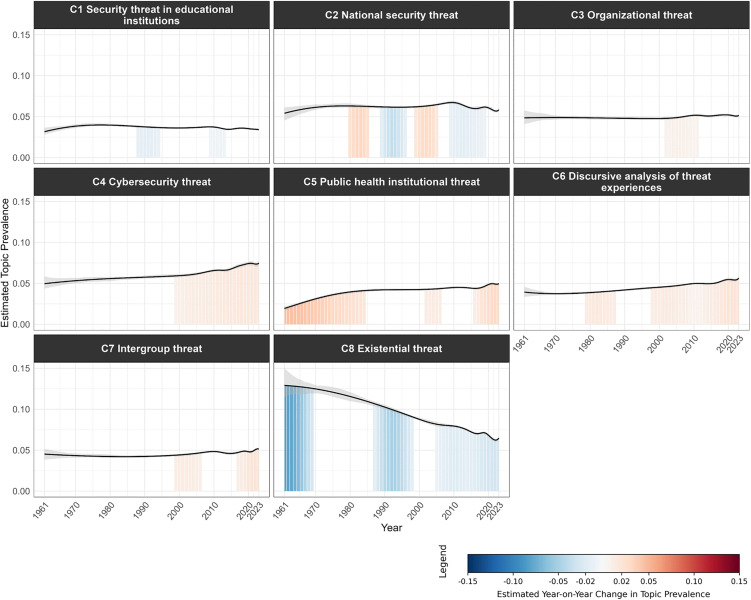
Temporal trends in topic prevalence (Cluster 4 Collective threats). The grey band represents the 95% confidence interval of the estimated topic prevalence. Shaded regions under the curve indicate periods of statistically significant year-on-year change in topic prevalence. Blue shading denotes a significant decrease, red shading a significant increase, and unshaded areas indicate non-significant change. Color intensity reflects the magnitude of change, with darker shades representing larger changes.

Figs 8–11 visualize the temporal trends for the four clusters, plotting trendlines for each topic’s prevalence by year and shaded regions under the curve to indicate years with significant changes in prevalence compared to the previous year (see S6 Fig for a consolidated heatmap of significant year-on-year prevalence change across all topics and clusters).

Both Cluster 1 (Fig 8) and Cluster 3 (Fig 10) generally declined in topic prevalence over time, with topic prevalences in recent years declining or remaining stable. Four topics in Cluster 1 spiked in prevalence during the 2000s to early 2010s, likely due to advancements in neuroimaging techniques (e.g., functional magnetic resonance imaging (fMRI), positron emission tomography (PET)) during the period, which enabled neural underpinnings of threat processing to be examined with unprecedented spatial precision and biological specificity [37,38]. For Cluster 3, S*1 Stereotype threat in academic contexts* spiked in prevalence during the late 1990s, following Steele and Aronson’s seminal publication on stereotype threat [39].

Cluster 2 (Fig 9) generally showed an early peak in prevalence followed by a subsequent decline. *H1 Illness threat and coping*, *H6 Risky health behaviors*, and *H7 Pandemic threat* showed a surge in recent years driven by the COVID-19 pandemic, which brought unprecedented attention to infectious disease threats, coping with illness and isolation, and health-related behaviors.

Cluster 4 (Fig 11) generally showed an increase in prevalence over time, tracking broader societal developments and global events. For instance, *C4 Cybersecurity threat* showed sustained growth in the post-2000s period. Given the widespread adoption of digital technologies across governments, organizations, and institutions in recent decades, the scope and severity of cybersecurity threats have rapidly expanded, driving research into digital security, data protection, and threat mitigation [40]. The increased prevalence of *C7 Intergroup threat* in the early 2000s and late 2010s was aligned with major geopolitical and sociopolitical events, including 9/11 terrorist attacks [41], rising global nationalism [42], and heightened racial tensions as highlighted by movements like Black Lives Matter [43].

#### Topics exhibiting substantial temporal changes in prevalence.

**Fig 12:**
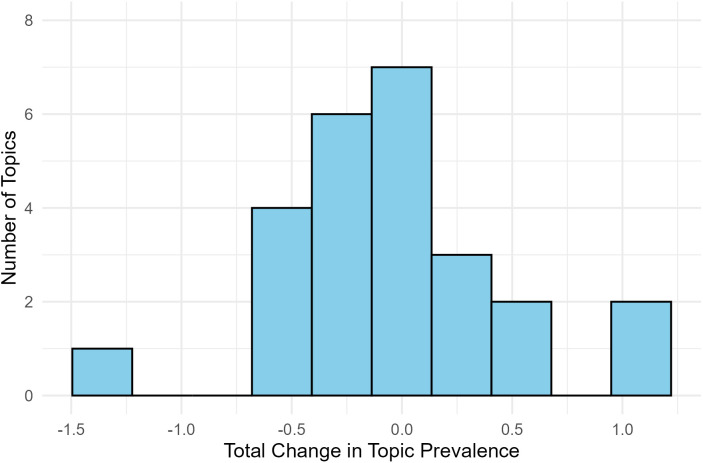
Distribution of topics by total change in topic prevalence (summed across years with significant change).

To identify topics with greatest shifts in prevalence, we summed significant year-on-year changes for each topic (mean = −0.058, *S.D.* = 0.514; see Fig 12 for distribution). Topics exceeding one standard deviation above or below the mean cumulative change were classified as showing substantial changes.

Two topics substantially increased in prevalence over time. *H7 Pandemic threat* (Fig 9) underwent episodic increases in periods with major disease outbreaks (SARS/H5N1 in early 2000s, Ebola/Zika in the late 2010s), with the most dramatic surge beginning in 2020 with the COVID-19 pandemic. *C5 Public health institutional threat* (Fig 11) had comparable periods of growth in the early 2000s and late 2010s but also showed an initial rise between the 1960s and 1980s coinciding with healthcare system expansion (e.g., Medicare, Medicaid) and HIV/AIDS institutional responses.

Three topics substantially declined in prevalence over time. Both *P2 Conditioned/reflexive physiological responses* (Fig 8) and *C8 Existential threat* (Fig 11) were initially highly prevalent but subsequently underwent steep decline, reflecting broader shifts in psychology away from behaviorist [44] and psychoanalytic or existential [45] frameworks that had dominated early decades [46]. *H5 Self/other harm in clinical contexts* (Fig 9) showed sustained decline starting in the late 1980s. The decline coincided with the deinstitutionalization movement’s shift from psychiatric hospitals to community-based mental healthcare, which likely reduced research focus on harm within institutional clinical contexts [47].

### Synthesis of key findings

Despite exponential growth in psychological threat research over past decades, systematic cross-field synthesis has remained absent. We addressed this gap by applying structural topic modeling on 51,903 publications gathered from APA PsycInfo, Scopus, and Web of Science to map the thematic structure, connectivity patterns, and temporal evolution.

The structural topic model revealed 25 key topics within the literature, clustered into four research domains primarily organized along disciplinary boundaries and levels of social organization. Notably, topics examining general psychological mechanisms comprised only a minority (24%), with most topics focused on specific threat types ranging from health and clinical, social psychological, to collective threats. Connections between research domains were sparse with no domain serving as a central hub, suggesting limited integration across the field. Crucially, threat processing mechanisms showed weak connectivity with both health and clinical threats and collective threats. These patterns indicate that research on fundamental psychological threat processes and domain-specific investigations operate in relative isolation, constraining both translation of basic findings and theory refinement. Research domains varied in internal cohesion. The collective threat domain exhibited especially weak cohesion, appearing to be grouped primarily by level of analysis. Temporal analyses revealed diversification of research attention over the past six decades, with cluster- and topic-level trajectories reflecting responsiveness to paradigm shifts (e.g., decline of behaviorism and psychoanalysis), methodological advances (e.g., neuroimaging), and especially societal events. The COVID-19 pandemic exemplified this responsiveness, catalyzing growth in pandemic threat research and related topics including illness threat and coping, risky health behaviors, and public health institutional threat.

Analyses of psychological literature on threat revealed a landscape of extensive research spanning diverse disciplinary perspectives and theoretical traditions. Yet, knowledge was organized primarily around specific threat manifestations rather than general psychological processes, posing challenges to cumulative knowledge-building and reflecting broader tensions between specialization and integration in psychological science [48,49]. Further, recent temporal patterns, namely declining attention to threat processing mechanisms alongside event-driven interest on health and collective threats, suggest that psychological science may be increasingly reactive rather than theory-driven in studying threat [50,51]. Reactivity ensures that psychological science engages with real-world issues and informs interventions, but has important limitations. For instance, although COVID-19 catalyzed substantial research [52,53], attention will inevitably wane until the next crisis. While research on specific threats may accumulate within silos, episodic attention hinders sustained theoretical development beyond crisis-specific applications. With decades of extensive research documented here, the field is well-positioned to move from domain-specific accumulation toward cross-field integration, developing frameworks that apply across threat types and inform responses to emerging challenges.

### Future directions

#### Construct clarity.

Researchers utilize the same term “threat” across investigations into different aspects and manifestations of threat, ranging from physical and psychological to social and existential. While the word “threat” can be intuitively understood, whether the term reflects a shared underlying construct or whether researchers are committing a jingle fallacy [54] is unclear. Evaluating whether the same term is being used to describe diverging constructs is critical for theoretical clarity and cumulative knowledge as integration efforts would otherwise risk conflating distinct psychological processes. As such, an essential step in advancing toward a unified and theoretically integrated psychology of threat is to first examine how threat is conceptualized across subfields and domains to clarify construct coherence and establish definitional boundaries.

#### General psychological mechanisms.

Analyses of network connectivity showed limited connectivity between threat processing mechanisms and other research domains. Threat processing mechanisms represent a strong potential unifying foundation within psychological literature on threat, as fundamental processes underlying threat detection, appraisal, and response should inform research on all specific threat manifestations. Future research should investigate the affective, behavioral, cognitive, and neural pathways activated across diverse threat domains, especially health and clinical contexts, to identify commonalities in psychological processing mechanisms and response patterns. In parallel, future research should also examine whether certain threat types necessitate distinct models due to unique contexts. For instance, responses to pathogen threats may involve disgust reactions [55], while existential threats may engage abstract cognitive processes such as worldview defense or symbolic affiliation [56]. Jonas et al. proposed a general process model of threat and defense (GPMTD) by drawing on social psychological research to identify common motivational processes underlying reactions to threats such as mortality salience, social exclusion, and identity threat [7]. While the GPMTD represents an important initial step toward identifying common psychological responses to threat, the model’s applicability to threats in other domains and the comprehensiveness of the proposed motivational processes remain uncertain. As the model is primarily grounded in social psychological research, threat experiences and mechanisms in other domains may not be adequately captured. Further work is needed to examine if core psychological processes underlie how threats are perceived, appraised, and acted upon across domains and to develop an integrative framework on threat processing and response with clarity on the extent of generalizability.

#### Financial threat.

While threats to group- and organizational-level material well-being were represented through the topics *C3 Organizational threat* and *C7 Intergroup threat*, threat to personal financial stability, which may arise from situations such as job loss, poverty, or larger economic crises, was not captured by any topic. Rather, topics examining threat at the individual-level primarily focused on physical and psychological well-being. Yet, financial threat has been found to have important implications for individuals’ physical and mental health [57,58]. A potential reason for this gap lies in the characteristics of the dominant research population in psychology, namely undergraduate samples from WEIRD (Western, Educated, Industrialized, Rich, and Democratic) societies [59]. These participants often experience relatively stable socioeconomic conditions and may have limited direct exposure to acute financial insecurity, possibly constraining the exploration of financial threat in psychological research. An alternative explanation is that while some psychological studies examine financial hardship, insecurity, or stress, these concepts are not always conceptualized or measured in terms of financial threat (i.e., perceived threat to one’s financial security). The absence of a dedicated topic in the model reflects a potential conceptual gap in the literature. By recognizing financial threat as a distinct and psychologically salient form of threat, future efforts can advance the understanding of how economic insecurity impacts well-being, especially in less privileged or more financially vulnerable populations.

#### Multi-level threats.

The four research domains examined threats at different levels of analysis. The structural hub *C5 Public health institutional threat*, which bridged Cluster 2 (Health and clinical threats) and Cluster 4 (Collective threats), highlights an important aspect of real-world threats – threats can operate simultaneously across multiple levels. For instance, the recent COVID-19 pandemic posed a threat not only to personal health but also broader existential threats to communities and humanity at large [60]. Yet, the limited connectivity between individual-level and collective-level domains suggests most studies examine threat at a single level of analysis. This gap may arise from the methodological tendency of psychological research to isolate constructs for the purposes of measurement and hypothesis testing [61,62]. Future research should examine how threats manifest and interact across levels, such as investigating how individual threat perceptions relate to collective responses, and how collective-level threats shape individual experiences. Such cross-level frameworks would better capture the complexity of real-world threat experiences and inform interventions operating at multiple levels.

### Limitations

#### Scope of inclusion.

As we limited our dataset to records from APA PsycInfo, Scopus, and Web of Science that contained the term “threat” in the title or abstract, the search may have introduced three forms of bias. Firstly, as our search strategy did not include synonyms for “threat”, our dataset may exclude studies that utilized synonymous terms such as “danger” or “risk”. These terms share some conceptual overlap with threat but are widely used across psychological literature to refer to distinct constructs and research areas like “danger” in hazard and safety perception, or “risk” in risk perception and decision-making. Given that including synonyms in the search strategy would have introduced considerable conceptual noise to the focal construct of threat, we restricted the search to the term “threat” to maintain conceptual specificity and coherence of the corpus. Secondly, our dataset may exclude relevant publications that investigated threat but did not explicitly use the term in the title or abstract and only mentioned the term in the main text. However, we expect the omission to be minimal as studies focused on threat typically reference the term in the title or abstract. Thirdly, our dataset may include publications that used the term “threat” in the title or abstract but did not examine threat. Due to the size of the dataset, a manual screening of all entries was not feasible. Nevertheless, the inclusion of the term in the record’s key fields suggests that “threat” should have been of conceptual relevance in most cases.

Additionally, our analysis was limited to records published in English. As structural topic modeling relies on word co-occurrence patterns to detect latent topics, including multilingual records would have introduced significant noise and complexity in the modeling process. Consequently, our dataset excludes psychological research on threat published in other languages and may underrepresent non-Western, culturally specific areas of threat investigation. Future efforts should explore multilingual text analysis methods to better capture global psychological research on threat.

#### Assessment of connections between thematic areas.

We assessed connections between thematic areas by examining the network structure of the topic co-occurrence network and leveraging shared APA descriptors in cross-cluster bridges. This approach provides insight into content-based associations and shared research focuses between thematic areas, but does not account for other forms of connections such as citation patterns or authorship networks. Future work could complement our approach by analyzing citation networks, journal metadata, or author collaboration patterns to develop a more comprehensive assessment of the extent to which thematic areas in the threat literature are interconnected through different types of scholarly relationships.

#### Temporal trend analyses.

We utilized two complementary approaches to examine topic prevalence trends over time as each method offered distinct advantages and limitations. The *stmprevalence* package offers uncertainty-aware visualization of topic prevalence trends by applying Bayesian beta regression, but does not support formal significance testing of changes over time. On the other hand, the *gratia* package fits generalized additive models to topic prevalence estimates and identifies periods of significant increase or decrease based on the first derivative of the fitted curve, but does not account for posterior uncertainty from the structural topic model. Given the lack of a single method for estimating significant changes in topic prevalence, the dual approach allowed us to triangulate findings and provide a nuanced account of temporal dynamics. Nevertheless, we acknowledge that the findings should be interpreted as exploratory. Future research would benefit from modeling frameworks that incorporate both model uncertainty estimates and significance testing to enhance the robustness and inferential validity of temporal topic prevalence analyses.

## Conclusion

The past six decades have yielded an expansive body of psychological knowledge on threat. The present study marks the first systematic attempt to map the landscape of research on threat across subfields through structural topic modeling and network analysis. Computational analyses of 51,903 publications revealed 25 key topics that clustered into four research domains primarily organized along disciplinary boundaries and levels of social organization, namely threat processing mechanisms, health and clinical threats, social psychological threats, and collective threats. The breadth of existing research showcases the diverse perspectives brought to bear on threat and underscores the rich insights gleaned through past scholarship. Yet, patterns of connections between research domains reflected limited integration across the field. Further, temporal trends in topic prevalence indicated diversification of research attention over time, with trajectories shaped by paradigm shifts, methodological advances, and societal events. Notably, declining attention to threat processing mechanisms alongside event-driven growth in health and collective threats suggests increasingly reactive rather than theory-driven inquiry.

As a core element of human cognition, emotion, and behavior, threat remains a construct of enduring relevance across psychological subfields. Continued research on threat within psychology is both inevitable and essential due to emerging real-world threats continually reshaping societal landscapes and the strong translational potential for informing interventions and policy. The next critical step, then, is to advance toward an integrated psychology of threat that bridges disciplinary boundaries and generates frameworks capable of capturing both the complexities of threat as a multifaceted psychological construct and commonalities in the psychological experience of threat across diverse manifestations.

## Supporting information

S1 Table Document types excluded from each database and record counts. (PDF)

S2 Table Text preprocessing steps and rationale. (PDF)

S3 Table Top FREX words for topics (K = 24, 25, 26). (PDF)

S4 Table Replication of cluster assignment across 1,000 bootstrapped iterations by topic. (PDF)

S5 Table Network centrality metrics by topic. (PDF)

S1 Fig Candidate model metrics by topic number. (TIF)

S2 Fig Topic exclusivity against semantic coherence (averaged across topics in each model). (TIF)

S3 Fig Topic exclusivity against semantic coherence (K = 26). (PNG)

S4 Fig Topic exclusivity against semantic coherence (K = 24). (PNG)

S5 Fig Topic exclusivity against semantic coherence (K = 25). (PNG)

S6 Fig Heatmap of years with significant change in topic prevalence across topics and clusters. The cells indicate whether the topic significantly increased (colored red) or decreased (colored blue) in each year compared to the previous year. Cell color intensity represents change magnitude, with darker shades representing greater changes. Grey cells represent years with no statistically significant changes in prevalence.
(TIF)

S1 File Selection of topic solution. (PDF)
