# Supplementary material for: Mapping the landscape of psychological literature on threat from 1961 to 2023 through structural topic modeling
Source: PLoS One. 2026 Jun 5;21(6):e0350996. doi: 10.1371/journal.pone.0350996 (PMC13240917; doi:10.1371/journal.pone.0350996)
Supplement: S1 Table — (PDF) [file pone.0350996.s001.pdf]

S1 Table. Document types excluded from each database and record counts.

Document Type	Count
APA PsycInfo	
Abstract Collection	3
Bibliography	8
Chapter; Comment/Reply	25
Chapter; Reprint	3
Column/Opinion	75
Comment/Reply	705
Comment/Reply; Reprint	1
Editorial	319
Encyclopedia	1
Encyclopedia Entry	4
Erratum/Correction	97
Interview	4
Journal Article; Reprint	1
Letter	154
Obituary	9
Retraction	2
Review-Book	543
Review-Media	15
Review-Software & Other	6
Scopus	
Conference review	3
Data paper	1
Editorial	88
Erratum	55
Letter	45
Note	142
Retracted	7
Web of Science	
Article; Data Paper	1
Article; Publication with Expression of Concern	1
Article; Retracted Publication	1
Bibliography	2
Biographical-Item	1
Book Review	62
Correction	32
Correction, Addition	1
Discussion	2
Editorial Material	404
Editorial Material; Book Chapter	13
Editorial Material; Early Access	4
Item About an Individual	1
Letter	24
Meeting Abstract	791
News Item	5
Note	65
