# Supplementary material for: Mapping the landscape of psychological literature on threat from 1961 to 2023 through structural topic modeling
Source: PLoS One. 2026 Jun 5;21(6):e0350996. doi: 10.1371/journal.pone.0350996 (PMC13240917; doi:10.1371/journal.pone.0350996)
Supplement: S2 Table — (PDF) [file pone.0350996.s002.pdf]

S2 Table. Text preprocessing steps and rationale.

Step	Description	Rationale
1. Lowercasing	Convert all text to lowercase	Ensures consistent matching of words regardless of case
2. URL removal	Remove hyperlinks	Eliminates non-semantic tokens that do not contribute to topic content
3. Punctuation cleaning	Remove punctuation (except hyphens)	Removes non-meaningful symbols
4. Hyphen normalization	Replace hyphens between words with underscores (e.g., "covid_19").	Retains compound expressions as single tokens
5. Whitespace normalization	Collapse repeated spaces into single spaces	Standardizes spacing for consistent tokenization
6. Tokenization	Split text into individual tokens using NLTK	Produces word-level input for subsequent steps
7. POS tagging and lemmatization	Assign part-of-speech (POS) tags and reduce inflected forms to their base lemmas (e.g., *running* → *run*)	Groups word variants under a common base form
8. Stopword removal	Remove common English stopwords (e.g., *the, and, of*) using NLTK's default list	Removes high-frequency function words that provide grammatical structure but little topical meaning
9. Short token removal	Exclude tokens shorter than three characters	Removes residual short tokens (e.g., *z*, *vs*) that reduce interpretability of topic-word outputs
