# Supplementary material for: Mapping the landscape of psychological literature on threat from 1961 to 2023 through structural topic modeling
Source: PLoS One. 2026 Jun 5;21(6):e0350996. doi: 10.1371/journal.pone.0350996 (PMC13240917; doi:10.1371/journal.pone.0350996)
Supplement: S3 Table — (PDF) [file pone.0350996.s003.pdf]

S3 Table. Top FREX words for topics (K = 24, 25, 26).

	Top ten FREX words		
**Topic**	**24-topic model**	**25-topic model**	**26-topic model**
1	stereotype, student, teacher, achievement, math, university, college, academic, school, performance	stereotype, performance, college, achievement, math, academic, student, university, feedback, test	stereotype, performance, college, math, student, achievement, academic, university, feedback, stem
2	law, officer, police, enforcement, justice, criminal, court, prison, gang, legal	police, law, officer, teacher, enforcement, school, justice, criminal, court, prison	police, officer, law, teacher, enforcement, justice, school, criminal, prison, gang
3	chapter, death, meaning, concept, dream, defense, author, describe, sense, mind	chapter, concept, dream, meaning, death, author, defense, mind, example, describe	chapter, death, concept, meaning, dream, author, mind, example, describe, defense
4	employee, organizational, firm, consumer, job, market, leadership, organization, product, industry	employee, organizational, firm, market, consumer, job, organization, manager, product, leadership	employee, organizational, firm, market, consumer, organization, job, manager, industry, product
5	parent, child, mother, adolescent, parental, childhood, youth, family, infant, adolescence	child, parent, mother, adolescent, parental, childhood, youth, adolescence, family, adversity	child, parent, mother, adolescent, parental, childhood, youth, family, adolescence, adversity
6	attachment, men, self_esteem, couple, male, aggressive, masculinity, romantic, narcissism, gender	attachment, men, male, romantic, masculinity, couple, narcissism, aggressive, jealousy, aggression	attachment, men, masculinity, romantic, male, aggressive, narcissism, couple, aggression, jealousy
7	covid_19, immigrant, prejudice, pandemic, attitude, chinese, religious, immigration, 2020, covid	covid_19, pandemic, climate, chinese, 2020, vaccine, uncertainty, covid, vaccination, china	covid_19, pandemic, climate, chinese, vaccine, 2020, covid, vaccination, china, outbreak
8	cancer, well_being, cop, resilience, mental, distress, sleep, illness, psychological, psychosocial	cancer, cop, well_being, distress, resilience, sleep, illness, breast, psychological, life	cancer, cop, well_being, distress, resilience, sleep, illness, breast, life, psychological
9	attentional, bias, amygdala, anxious, anxiety, cortex, neural, brain, word, prefrontal	anxiety, amygdala, anxious, word, cortex, neural, functional, brain, prefrontal, activation	bias, anxiety, attentional, anxious, word, attention, panic, interpretation, worry, stroop
10	pain, appraisal, physiological, stress, stressor, cortisol, heart, cardiovascular, reactivity, stressful	pain, stressor, stress, appraisal, cortisol, physiological, heart, cardiovascular, stressful, reactivity	pain, stressor, stress, appraisal, cortisol, physiological, heart, stressful, cardiovascular, reactivity
11	social, ingroup, group, member, collective, exclusion, identification, identity, moral, in_group	social, self_esteem, exclusion, collective, ingroup, rejection, identity, group, member, ostracism	social, self_esteem, exclusion, ingroup, collective, rejection, member, identity, group, ostracism
12	terrorism, war, terrorist, nuclear, international, military, book, world, psychology, modern	terrorism, war, terrorist, nuclear, international, book, military, society, century, politics	terrorism, war, terrorist, nuclear, international, book, moral, military, society, century
13	violence, victim, assault, ipv, abuse, sexual, victimization, rape, perpetrator, harassment	victim, ipv, assault, violence, victimization, abuse, sexual, rape, perpetrator, harassment	victim, ipv, assault, violence, victimization, abuse, sexual, rape, perpetrator, harassment
14	technology, privacy, security, internet, application, user, tool, online, systematic, digital	technology, security, privacy, application, user, internet, tool, digital, systematic, review	technology, security, privacy, tool, application, user, internet, digital, systematic, review

15	intention, message, motivation, efficacy, choice, feedback, experiment, behavior, appeal, preference	message, intention, motivation, efficacy, choice, decision, preference, appeal, judgment, reactance	message, intention, motivation, efficacy, choice, decision, preference, judgment, appeal, reactance
16	visual, animal, extinction, cue, picture, startle, aversive, memory, signal, conditioning	animal, extinction, startle, shock, conditioning, aversive, predator, signal, defensive, generalization[b]	animal, defensive, predator, specie, error, detection, detect, experiment, visual, accuracy[b]
17	suicide, nurse, hospital, suicidal, client, therapy, treatment, patient, physician, medical	suicide, nurse, suicidal, hospital, therapy, treatment, client, patient, physician, psychiatric	suicide, hospital, suicidal, nurse, patient, client, physician, treatment, psychiatric, therapy
18	program, service, climate, barrier, healthcare, food, health, disaster, intervention, obesity	service, healthcare, health, barrier, program, obesity, intervention, disaster, provider, emergency	service, healthcare, health, program, barrier, intervention, disaster, obesity, provider, emergency
19	culture, narrative, discourse, cultural, language, construction, south, space, frame, lesbian	theme, narrative, qualitative, discourse, language, construction, interview, space, in_depth, culture	theme, narrative, qualitative, discourse, language, interview, construction, in_depth, thematic, space
20	ptsd, symptom, scale, posttraumatic, depression, depressive, trauma, score, traumatic, ocd	ptsd, scale, symptom, posttraumatic, depression, depressive, trauma, score, traumatic, item	ptsd, scale, symptom, posttraumatic, depression, depressive, trauma, score, traumatic, ocd
21	emotion, expression, facial, avoidance, anger, emotional, angry, face, disgust, fear[a]	emotion, avoidance, affective, anger, sensitivity, disgust, fear, negative, implicit, reward[a]	emotion, anger, negative, disgust, implicit, sensitivity, positive, affective, reward, regulation
22	behaviour, personality, worry, shame, cognition, cognitive, thought, style, belief, trait	personality, behaviour, shame, worry, cognition, cognitive, loneliness, style, thought, trait	personality, behaviour, shame, schizophrenia, behavioural, loneliness, paranoia, self, psychosis, compassion
23	hiv, risk, old, alcohol, aid, drug, infection, driver, vaccine, vaccination	hiv, risk, drug, old, aid, alcohol, driver, respondent, adult, age	hiv, risk, old, drug, aid, alcohol, adult, respondent, age, prevalence
24	white, black, racial, neighborhood, resident, race, 2000, discrimination, hate, class	immigrant, racial, ethnic, prejudice, white, black, minority, immigration, american, race	immigrant, racial, ethnic, prejudice, white, black, minority, immigration, american, race
25		facial, angry, neutral, visual, fearful, expression, attention, face, eye, picture[a]	facial, angry, amygdala, cortex, brain, neural, happy, fearful, prefrontal, connectivity
26			fear, extinction, conditioning, memory, startle, generalization, aversive, learn, expectancy, learning[b]

[a]Orange cells indicate the topic introduced in the 25-topic solution and the topic from which it split in the 24-topic solution.

[b]Green cells indicate the topic introduced in the 26-topic solution and the topic from which it split in the 25-topic solution.
