# Supplementary material for: Mapping the landscape of psychological literature on threat from 1961 to 2023 through structural topic modeling
Source: PLoS One. 2026 Jun 5;21(6):e0350996. doi: 10.1371/journal.pone.0350996 (PMC13240917; doi:10.1371/journal.pone.0350996)
Supplement: S4 Table — (PDF) [file pone.0350996.s004.pdf]

S4 Table. Replication of cluster assignment across 1,000 bootstrapped iterations by topic.

Topic	Proportion Replicated	No. of iterations assigned to each cluster				
		Cluster 1	Cluster 2	Cluster 3	Cluster 4	Cluster 5[a]
P1	1	1000	0	0	0	0
P2	1	1000	0	0	0	0
P3	1	1000	0	0	0	0
P4	0.998	998	2	0	0	0
P5	0.995	995	0	5	0	0
P6	0.873	873	127	0	0	0
H1	0.808	188	808	0	4	0
H2	0.798	202	798	0	0	0
H3	0.797	181	797	5	17	0
H4	0.787	16	787	0	196	1
H5	0.763	19	763	0	216	2
H6	0.754	0	754	6	237	3
H7	0.723	0	723	162	112	3
S1	0.955	40	0	955	5	0
S2	0.955	40	0	955	5	0
S3	0.955	39	0	955	6	0
S4	0.937	31	1	937	29	2
C1	0.996	0	4	0	996	0
C2	0.995	0	2	3	995	0
C3	0.987	0	4	6	987	3
C4	0.986	0	5	6	986	3
C5	0.946	0	45	6	946	3
C6	0.862	0	1	137	862	0
C7	0.761	0	0	239	761	0
C8	0.695	112	4	187	695	2

[a]The community detection algorithm occasionally identified a five-cluster solution in a small minority (0.3%) of the 1,000 bootstrap iterations. A three-cluster solution was identified in 23.90% of the iterations, and a four-cluster solution in 75.80% of the iterations.
