# Supplementary material for: Mapping the landscape of psychological literature on threat from 1961 to 2023 through structural topic modeling
Source: PLoS One. 2026 Jun 5;21(6):e0350996. doi: 10.1371/journal.pone.0350996 (PMC13240917; doi:10.1371/journal.pone.0350996)
Supplement: S5 Table — (PDF) [file pone.0350996.s005.pdf]

S5 Table. Network centrality metrics by topic.

Topic	Strength Centrality	Betweenness Centrality	Closeness Centrality	Eigenvector Centrality
P1	1.61	0.00	0.10	0.48
P2	0.97	0.00	0.09	0.28
P3	1.13	0.00	0.10	0.36
P4	1.51	0.00	0.12	0.55
**P5**[a]	2.79	0.27	0.13	0.82
P6	0.72	0.00	0.08	0.21
H1	1.43	0.01	0.09	0.33
H2	1.35	0.05	0.09	0.28
H3	0.80	0.00	0.07	0.16
H4	0.62	0.00	0.08	0.12
H5	1.38	0.15	0.12	0.46
H6	1.82	0.12	0.11	0.41
H7	0.64	0.00	0.08	0.20
S1	0.59	0.00	0.08	0.17
S2	0.90	0.00	0.09	0.30
S3	1.88	0.13	0.12	0.61
S4	1.72	0.02	0.12	0.58
C1	0.74	0.00	0.10	0.27
C2	1.82	0.09	0.13	0.63
C3	0.80	0.00	0.10	0.29
C4	1.56	0.01	0.12	0.53
**C5**[a]	2.50	0.17	0.13	0.71
C6	1.86	0.11	0.13	0.67
C7	0.66	0.00	0.10	0.25
**C8**[a]	3.01	0.39	0.15	1.00

[a]Bolded topics indicate structural hubs (i.e., nodes with relatively high centrality across multiple indices).
