# Supplementary figures and images for: Mapping the landscape of psychological literature on threat from 1961 to 2023 through structural topic modeling
Source: PLoS One. 2026 Jun 5;21(6):e0350996. doi: 10.1371/journal.pone.0350996 (PMC13240917; doi:10.1371/journal.pone.0350996)

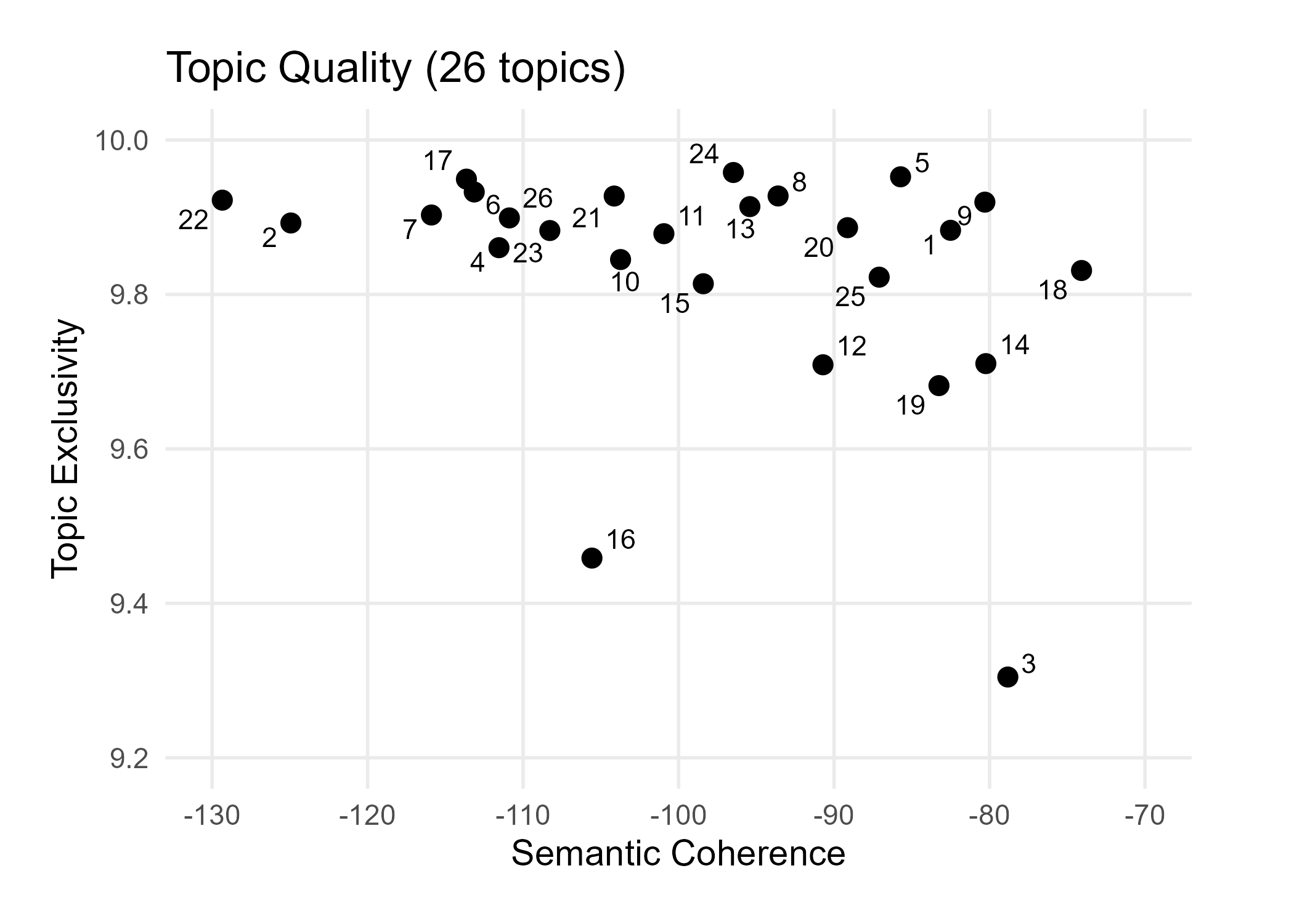

Supplement: S3 Fig — (PNG) [file pone.0350996.s008.png]

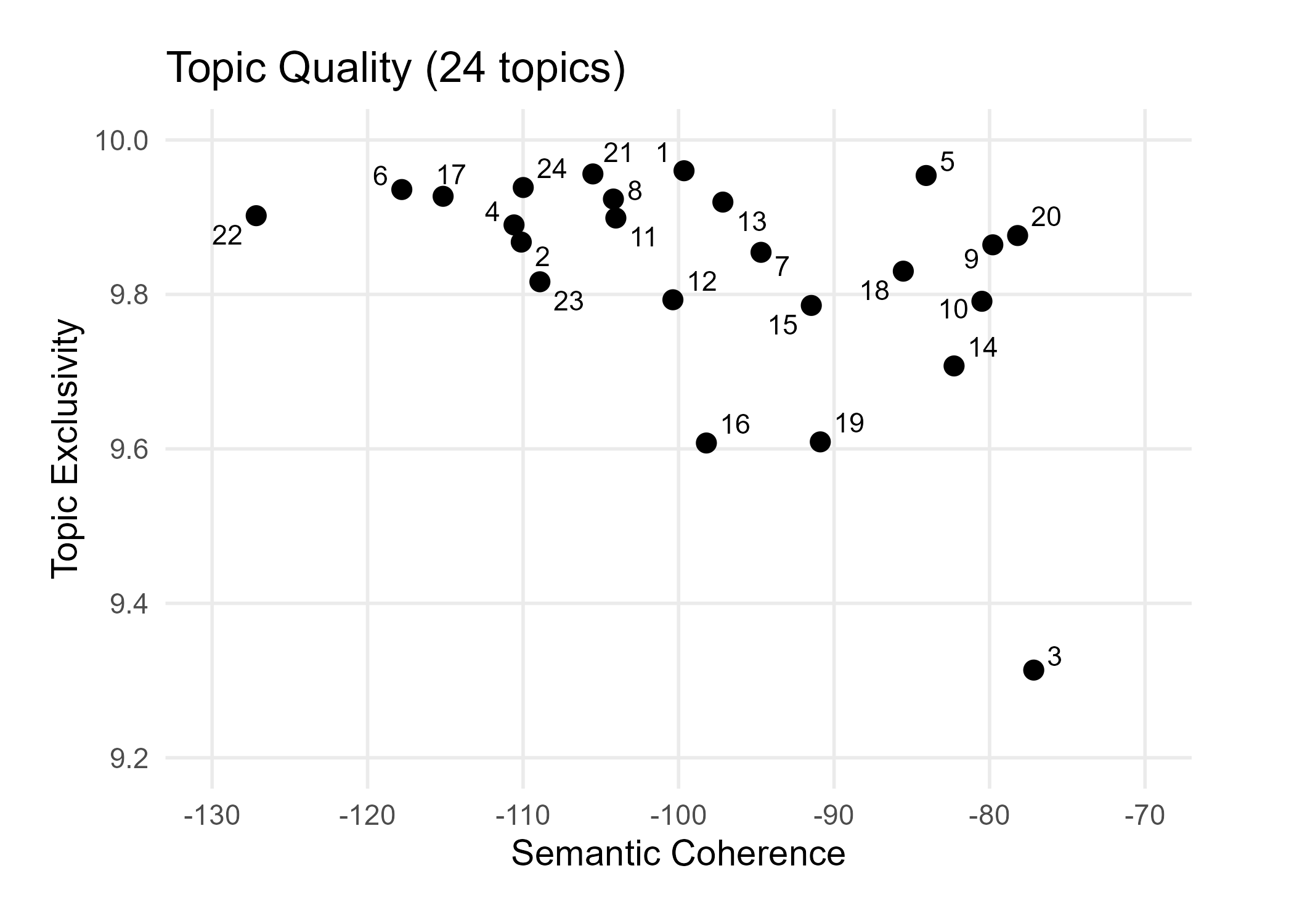

Supplement: S4 Fig — (PNG) [file pone.0350996.s009.png]

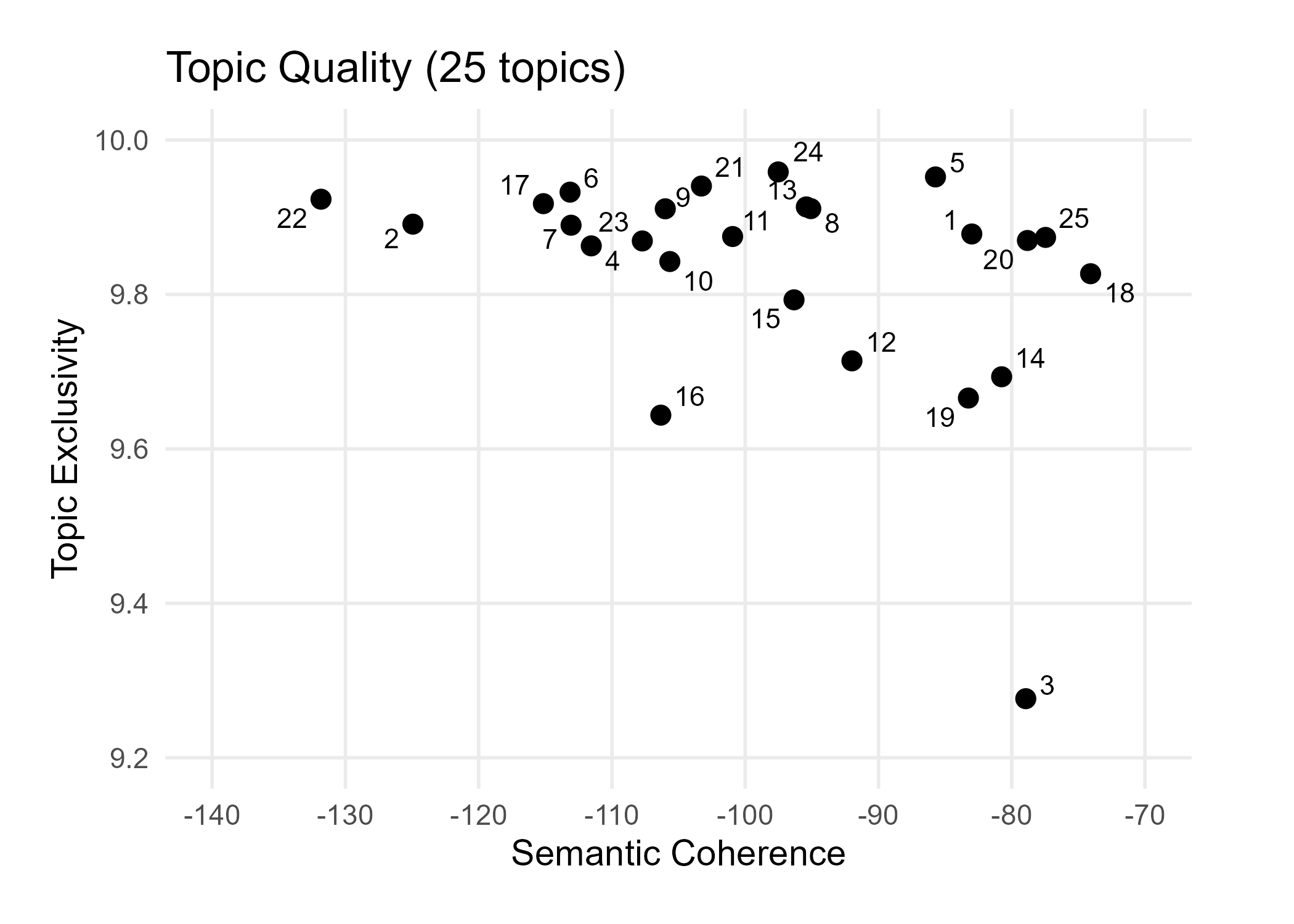

Supplement: S5 Fig — (PNG) [file pone.0350996.s010.png]
