# Supplementary material for: Mapping the landscape of psychological literature on threat from 1961 to 2023 through structural topic modeling
Source: PLoS One. 2026 Jun 5;21(6):e0350996. doi: 10.1371/journal.pone.0350996 (PMC13240917; doi:10.1371/journal.pone.0350996)
Supplement: S1 File — (PDF) [file pone.0350996.s012.pdf]

S1 File. Selection of topic solution.

Similar to conventional topic modeling with latent Dirichlet allocation (LDA), the optimal number of topics is not automatically determined in structural topic modeling. Rather, researchers typically explore a range of potential topic numbers and select the final model through a combination of quantitative and qualitative evaluations [1,2]. A good topic model should fit the data well while producing distinct and interpretable topics, such that each topic captures a coherent set of words representing a clear theme within the dataset. Quantitative metrics of model performance are used to assess model fit (e.g., perplexity, residuals) and topic quality (e.g., semantic coherence, exclusivity). However, quantitative metrics alone cannot fully assess the interpretability and meaningfulness of topics, which are best evaluated qualitatively. Since qualitative evaluation is time-consuming, researchers would typically first shortlist potential topic solutions using quantitative metrics. Subsequently, researchers would qualitatively assess topic interpretability and distinctiveness by evaluating the top words associated with each topic in the shortlisted topic solutions to select the final topic model.

We generated candidate models ranging from five to 100 topics, in intervals of five. S1 Fig shows the distribution of model fit metrics (held-out likelihood, lower bound, residuals) and topic quality (semantic coherence) over the candidate models. We identified the 20- to 30-topics window for further evaluation due to the balance between model fit and topic quality.

S1 Fig. Candidate model metrics by topic number.

We generated candidate models from 20 to 30 topics, in intervals of one. S2 Fig plots topic exclusivity against semantic coherence for each candidate model. Each point represents one model, with values averaged across all topics within that model. We shortlisted $K = 24$,

25, and 26 as the models demonstrated good balance between exclusivity and coherence, indicating that topics were distinct yet coherent.

S2 Fig. Topic exclusivity against semantic coherence (averaged across topics in each model).

For the three shortlisted models, we plotted semantic coherence and exclusivity scores of each topic within the model to conduct a finer-grained assessment of topic quality. As shown in S3 Fig, the 26-topic model displayed lower exclusivity for Topic 16, suggesting possible topic splitting and reduced interpretability. In contrast, the 24- (S4 Fig) and 25-topic (S5 Fig) models demonstrated a more stable balance between coherence and exclusivity, indicating that topics were both distinct and semantically consistent.

S3 Fig. Topic exclusivity against semantic coherence (K = 26).

S4 Fig. Topic exclusivity against semantic coherence (K = 24).

S5 Fig. Topic exclusivity against semantic coherence (K = 25).

We also evaluated the interpretability of the topics within the three models by reviewing top FREX words (S3 Table). In the 24-topic solution, Topic 7 encompassed terms related to both COVID-19 and prejudice toward immigrants. In contrast, the 25- and 26-topic solutions yielded a more focused version of this topic with top FREX words predominantly referring to the COVID-19 outbreak, improving thematic clarity. Comparing 24-topic and 25-topic solutions, Topic 25 likely split from Topic 21, with the 25-topic solution separating affective–motivational processing (Topic 21) from attentional processes toward threatening faces (Topic 25). Comparing 25-topic and 26-topic solutions, Topic 26 likely split from Topic 16, with the 26-topic solution separating research on animal models (Topic 16) from learning and conditioning (Topic 26). However, Topic 16 showed poorer topic exclusivity in the 26-topic solution compared to 25-topic solution, indicating that the additional topic may have fragmented an otherwise coherent theme rather than improving differentiation.

We selected the 25-topic model as the final solution as the model provided the best trade-off between semantic coherence and exclusivity across topics and produced the most interpretable structure. Compared to the 24-topic model, the 25-topic model yielded finer thematic differentiation, and unlike the 26-topic model, did not introduce redundant or fragmented topics.

References

1. Roberts ME, Stewart BM, Tingley D, Lucas C, Leder-Luis J, Gadarian SK, et al. Structural topic models for open-ended survey responses. American Journal of Political Science. 2014;58(4):1064–82.
2. Wallach HM, Murray I, Salakhutdinov R, Mimno D. Evaluation methods for topic models. Proceedings of the 26th Annual International Conference on Machine Learning. Montreal; 2009.
